# Soil microbial adaptation to carbon deprivation: shifts in lignocellulolytic gene profiles following long-term plant exclusion

**DOI:** 10.1186/s40793-025-00810-6

**Published:** 2025-12-10

**Authors:** David B. Fidler, Paul B. L. George, Lucas J. Le Brun, Robert I. Griffiths, Davey L. Jones, James E. McDonald

**Affiliations:** 1 Environment Centre Wales, School of Environmental and Natural Sciences, Bangor University, Gwynedd, LL57 2UW UK; 2 Département de Biochimie, de Microbiologie et de Bio-Informatique, Université Laval, Quebec City, QC G1V 0A6 Canada; 3 School of Biosciences, Birmingham Institute of Forest Research, Institute of Microbiology and Infection, University of Birmingham, Birmingham, B15 2TT UK

**Keywords:** Carbohydrate-active enzymes, CAZymes, Cellulases, Lignocellulose degradation, Metagenomics, Metabolomics, Soil carbon cycling, Xylanases

## Abstract

**Background:**

Lignocellulose represents a primary input of organic carbon (C) into soils, yet the identity of specific microorganisms and genes which drive lignocellulose turnover in soils remains poorly understood. To address this knowledge gap, we used a 10-year grassland plant-exclusion experiment to investigate how reduced plant C inputs affect microbial communities and their lignocellulolytic potential using a combination of metagenomic sequencing and untargeted metabolomics. We specifically tested the hypothesis that microbial community function in bare fallow plots would transition towards microbiota with genes for recalcitrant biomass degradation (i.e., lignocellulose), when compared to grassland plots with high labile C inputs.

**Results:**

Long-term plant exclusion lowered soil C and nitrogen (N) and reduced cellulose content, whilst hemicellulose and lignin were unchanged. Similarly soil microbiomes were highly distinct in long-term bare soils, along with soil extracellular enzyme profiles, though short-term plant-removal effects were less apparent. Plant exclusion resulted in a general enrichment of Firmicutes, Thaumarchaeota, Acidobacteria, Fusobacteria, and Ascomycota, with a general reduction in Actinobacteria. However, changes in bare soil lignocellulose degradation genes were more associated with discrete taxa from diverse lineages, particularly the Proteobacteria. Grouping of lignocellulose-degrading genes into broad substrate classes (cellulases, hemicellulases and lignases) revealed a possible increase in lignin degradation genes under plant exclusion confirming our hypothesis, although all other changes were at the level of the carbohydrate-active enzyme (CAZy) family. Intriguingly, untargeted metabolome profiles were highly responsive to plant exclusion, even after only one year. Bare soils were depleted in oligosaccharides and enriched in monosaccharides, fatty and carboxylic acids, supporting emerging evidence of long-term persistent C being within simple compounds.

**Conclusions:**

Together our data show that extracellular lignin degrading enzymes increase under long-term plant exclusion. There is now a need for increased focus on the microbial metabolic mechanisms which regulate the processing and persistence of enzymatically released compounds, particularly in energy limited soils.

**Graphical abstract:**

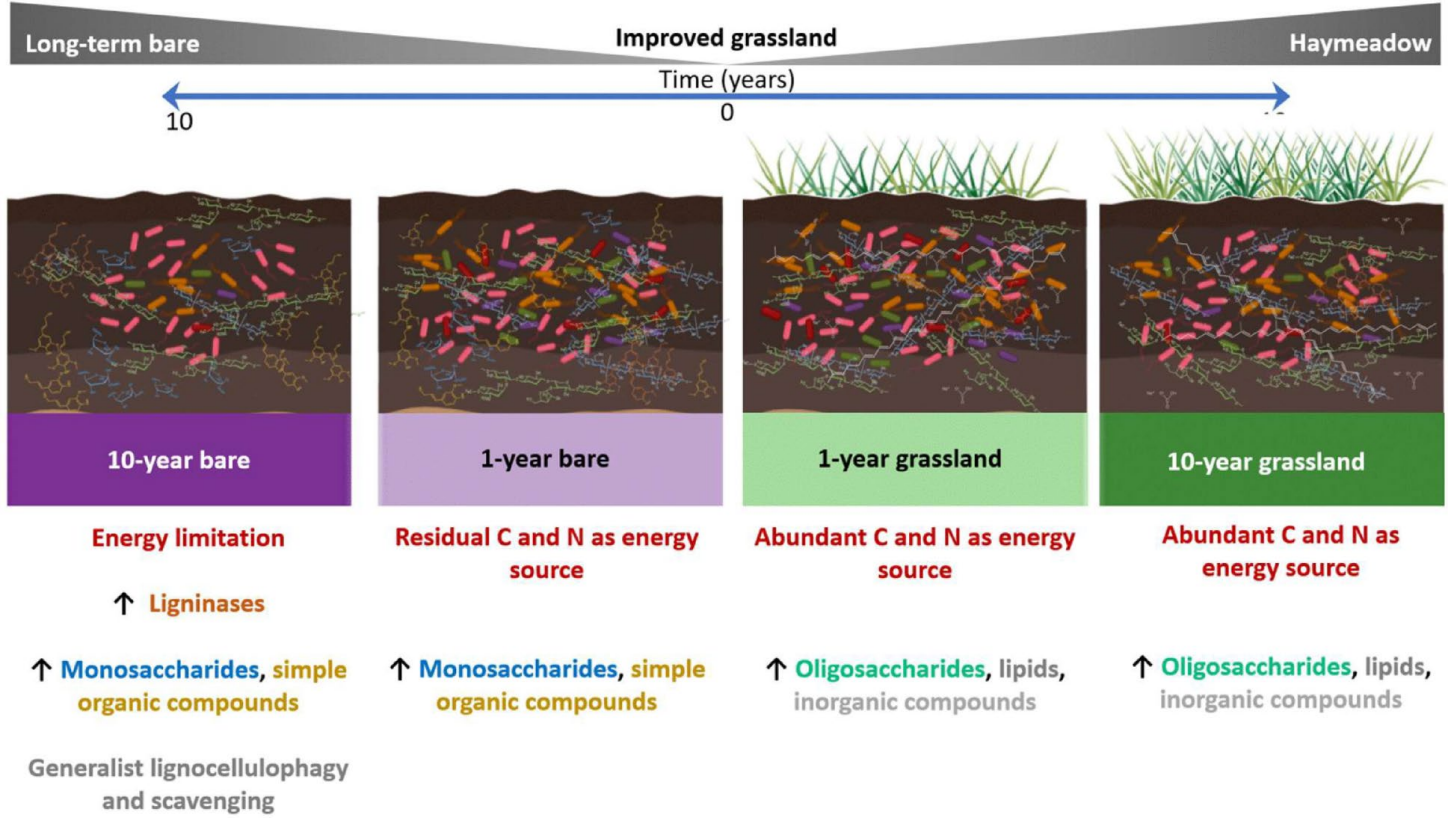

**Supplementary Information:**

The online version contains supplementary material available at 10.1186/s40793-025-00810-6.

## Background

Globally, grasslands store 34% of terrestrial carbon (C), cover 26% of land area, and account for 80% of agriculturally productive land [1]. The conversion of grasslands to arable land dramatically alters soil C dynamics, with grasslands acting as effective C sinks, while arable land serves as a major C source. This is most easily evidenced by the 25–75% reduction in soil organic C (SOC) in arable soils relative to natural grasslands [2]. Microorganisms have evolved diverse genomic strategies for the decomposition of insoluble lignocellulosic plant biomass, which (alongside other mechanical mechanisms) drive these reductions in SOC in arable land [2]. This genomic machinery is widely distributed across bacterial and fungal taxa, and underpins multiple global nutrient cycles [3]. However, relatively few studies have linked the oxidative and hydrolytic enzymes to the organisms which produce them. Such linkages are key to understanding how environmental perturbations may affect ecosystem functioning. They also enable targeted analysis of enzymes or microbiota with potential biotechnological applications, providing information to improve the next generation of C cycling models.

Metagenomic approaches have been used to link taxonomy to lignocellulolytic function in forest and peat soils [1, 4–10], however these relationships remain unstudied in grasslands, representing a key knowledge gap for understanding the interactions of microorganisms and soil C storage. More generally, long-term bare-fallow soils from across Europe have been used as valuable experimental systems to investigate the decomposition of soil organic matter (SOM), showing that in the first few decades, the rate of SOM decomposition is proportional to litter quality [11]. However, over time, plant-derived inputs decrease and microorganism-derived compounds increase [12]. As a result, the rate of SOM depletion becomes dependent on the metabolic rate of microbial decomposers and the rate of decrease in C quality [11]. Previous studies have shown that long-term bare-fallow soils maintain the capacity to mineralise cellulose and straw [13]. These soils have increased proportions of Actinomycetes and Fungi, reduced SOM content, microbial biomass, and mineralisation rates of insoluble C substrates relative to grassland soils [14]. However, limited research has been conducted on the microbial composition of these useful experimental systems, particularly with respect to the genetic control of lignocellulose turnover in soil.

To address this key knowledge gap in our mechanistic understanding of soil C cycling, we investigated the taxonomic and functional diversity of microorganisms responsible for lignocellulosic plant biomass turnover in grassland soils. To achieve this, we employed a long-term C deprivation experiment (grassland *vs* bare plots), integrating a combination of lignocellulose analysis, elemental analysis, metabolomics, and metagenomics. By comparing grassland *vs* bare treatments, we sought to determine their effects on soil chemical characteristics, such as the abundance of lignocellulose and its breakdown products. We also examined microbial community composition and gene families associated with lignocellulose turnover in soils (here referred to as: (1) any lignocellulolytic activity: carbohydrate-active enzymes (CAZy; CAZymes), lignocellulolytic genes, (2) cellulolytic activity: cellulase genes, (3) hemicellulolytic activity: hemicellulase genes, xylanase genes, and (4) lignin degradation: ligninase genes, auxiliary activity (AA) genes). We hypothesised that microbial community function in bare fallow plots would transition towards microbiota with genes for recalcitrant biomass degradation (i.e., lignocellulose), when compared to grassland plots with high labile C inputs.

## Methods

### Experimental design

Soils for this experiment were collected from Bangor University’s Henfaes Research Centre, Abergwyngregyn, UK (53.24° N, 4.02° W; 12 m a.s.l.). The experiment consisted of two plot age categories: six 9 m^2^ plots established in 2005 (henceforth “10-year”), and a further eight plots (henceforth “1-year”) established in 2015 adjacent to the 10-year plots to increase replication and to explore temporal relationships with plant exclusion. This gave a total of fourteen plots. Half of the plots in each age category (three in 10-year and four in 1-year) were covered in two layers of black, gas- and water-permeable fabric (henceforth “bare”) to prevent plant growth, while the remaining plots served as controls (henceforth “grassland”) and were mown annually and the harvested grass removed (Figs. S1 and S2). Each plot was demarcated by plastic frames inserted 25 cm into the soil, with 5–8 cm protruding above ground. TDR temperature sensors coupled to SDI-12 data loggers (Acclima Inc., Meridian, ID) showed that the black fabric did not alter the daily average soil temperature (*p* > 0.05). Prior to plot establishment the field was used for sheep grazing, and upon establishment, the plots were allowed to naturally revegetate. The site has a mean annual soil temperature at 10 cm of 10.2 °C, a mean annual rainfall of 1060 mm and has a temperate oceanic climate regime. The soil is classified as a sandy clay loam textured Eutric Cambisol with crumb structure (sand: 49% (standard error (SE) = 2%), silt: 31.3% (SE = 0.9%), clay: 19.7% (SE = 1.2%)), pH of 5.04 (SE = 0.04), with 78 (SE = 40) g SOM kg^−1^, 31.1 (SE = 1.6) g total C kg^−1^, and total nitrogen (N) of 3.1 (SE = 0.1) g total N kg^−1^ of dry soil). The vegetation consists largely of *Lolium perenne* L. interspersed with *Holcus lanatus* L. and *Festuca ovina* L. Ten subsamples of soil (mixed bulk and rhizosphere in the grassland, bulk only in the bare as no rhizosphere should exist) were collected from each plot (*n* = 14 plots) in spring 2015 and 2016 using a 1 cm diameter stainless steel soil corer (0 – 10 cm depth), for the 2005 and 2015 plots, respectively. Subsamples from each plot were homogenised and pooled. Each sample was subsampled and either air-dried for physicochemical analysis, immediately frozen (−80 °C) and freeze-dried for metabolomic analyses, or transferred to a − 80 °C freezer for DNA extraction. A detailed table of soil properties for the samples can be found in Table 1 of George et al. [15]. Given the long-term nature of the experiment, further replication was not possible.

### Organic matter analysis and total C

To measure total soil C, frozen soil was oven-dried at 105 °C, ground to pass a 2 mm sieve, and analysed using a TruSpec CN (Leco Corp, St Joseph, MI). For lignocellulose analysis, frozen soils were oven-dried at 40 °C for 24 h, and 0.5 g of soil placed into an Ankom F57 fibre filter bag (ANKOM Technology, Macedon NY, USA) which was then closed by heat-sealing. Neutral detergent fibre (NDF) and acid detergent fibre (ADF) procedures were performed sequentially on an Ankom 2000 analyser following the manufacturer’s instructions. After the NDF and ADF cycles, the filter bags were washed in acetone for 5 min and were oven dried at 105 °C for 4 h before being weighed. Lignin content was measured (sequentially) via acid detergent lignin (ADL) in a daisy^II^ incubator (Ankom) for 3 h, following the manufacturer’s instructions. Samples were left to air dry, before being oven dried at 105 °C for 4 h; each sample was then weighed. Ash content of the samples was determined through combustion in a Carbolite CWF 1200 muffle furnace (Carbolite, Hope Valley, UK) at 525 °C for 3 h. Samples were then weighed. The proportions of cellulose, hemicellulose, and lignin in each sample was calculated as described in Baker et al. [16] and are presented as grams per kilogram of dry soil.

### Metabolomics

Untargeted metabolomics for untargeted primary metabolites was performed by The West Coast Metabolomics Center (UC Davis Genome Center, Davis, CA, USA) by automated liner exchange cold injection system gas chromatography time of flight mass spectrometry (ALEX-CIS GCTOF MS) as described elsewhere [17].

The metabolomics data were pre-processed using ChromaTOF (v2.34; Leco Corp.). Briefly, baseline subtraction was applied just above the noise level and automatic mass spectral deconvolution and peak detection was applied at a 5:1 signal-to-noise ratio throughout the chromatogram. The BinBase rtx5 algorithm was applied, and spectra were cut to 5% base peak abundance and matched to database entries. Unmatched peaks were entered as new database entries when the signal-to-noise ratio was > 25 and purity < 1.0.

### DNA extraction and sequencing

DNA was extracted from the frozen soil samples, following a CTAB/Phenol Chloroform-based extraction method [18] but with RNAse A treatment prior to PEG precipitation (6 μL RNAse A 10 mg/mL (ThermoFisher Scientific, Waltham, MA, US)) of the DNA, incubation at 37 °C for 30 min, followed by an additional chloroform/isoamyl alcohol wash). A blank sample was included to act as a negative process control. All samples and the negative control were sent for library preparation (TruSeq Nano kit 350 bp inserts; Illumina, Cambridge, UK) and paired-end sequencing (single lane of an Illumina HiSeq 4000, 2 × 150 bp) at the Centre for Genomic Research, Liverpool University.

### Bioinformatics

#### Assembly

Sequence reads underwent quality control as follows: adapter sequences were trimmed using Cutadapt 1.2.1 [19] with -O 3. Sickle 1.200 was used to quality-trim the files, using a minimum window phred score of 20 [20]. Reads shorter than 20 bp were removed. Sequence quality was checked using fastq-stats from EAUtils [21]. For the assembly of all sequences in the metagenome, each library was dereplicated using prinseq-lite 0.20.4 with ‘-derep 1’ [22]. Dereplicated reads were then co-assembled using MEGAHIT 1.1.3 using default settings [23]. Basic assembly statistics were checked using Metaquast-5.0.0 [24].

#### Lignocellulase gene prediction

Open reading frames and translated protein sequences were predicted from the co-assembly using Prodigal 2.6.3 using “-p meta” and “-a” options [25]. The reads for each sample were mapped back to the assembly using bowtie2 2.3.4.3 using a seed of 1 [26]. The resulting SAM files were converted to sorted BAM files using SAMtools 1.9 [27]. Reads mapping to predicted gene sequences were counted for each sample using featureCounts 1.6.3 [28], with options “-P”, “-f”, “-B”, and “-C”. Feature type counted was “CDS”, and the gene identifier column was “ID”. Carbohydrate-active enzyme sequences in the assembly were identified using the dbCAN2 pipeline (using the databases CAZyDB.07312018.fa, and dbCAN-HMMdb-V7.txt), and only sequences genes with a signal peptide (identified using signalP 6.0 g) were kept for further analysis [29, 30]. The rule for assigning CAZy domain identity where tools did not concur was priority for the HMMER annotation over the DIAMOND annotation over the Hotpep annotation. To capture genes involved in lignocelluolysis we a priori chose to focus on CAZy families with high proportions of genes that have been shown to cause, or be involved in, the breakdown of specific lignocellulosic polymers [31]; these were: (i) cellulases: GH5, GH6, GH7, GH8, GH9, GH12, GH44, GH45, GH48; (ii) xylanases: GH10, GH11, GH8, GH30; (iii) lytic polysaccharide monooxygenases (LPMOs): AA9, AA10, AA11, AA13, AA14, AA15, (iv) all CBM families, and (v) all other AA families. All subfamilies of these CAZy families were included in the analysis. These genes are henceforth referred to as ‘lignocellulase genes’.

#### Taxonomic annotation of contigs

Kraken2 [32], Kaiju 1.6.3 [33] and CLARK v1.2.6 [34] were used to assign taxonomy to contigs, searching against genomes from *Bacteria*, *Archaea*, *Protozoa*, and *Fungi*, on RefSeq release 93 [35]. Final taxonomic assignment followed the rule Kraken2 > Kaiju > CLARK [32, 36]. SAMtools faidx and BEDtools genomeCoverageBed [37] were used to count reads mapping to each contig from each sample. Contigs found in the negative control library were removed from all analyses. Contig level counts per million (CPM) were calculated from fragments per kilobase million values from pileup.sh from bbtools [38]. Contigs that could not be assigned to a phylum were not included in analyses of community composition or CAZyme origins.

#### KEGG annotation of contigs and metabolites

Functional genes were assigned to contigs using MetaCerberus 1.3.2 [39] from the translated protein sequences predicted by Prodigal. Annotation was performed against KOfam, a Hidden Markov Models (HMM) database of KEGG Orthologs [40], using the options “- protein” and “- hmm KOFam_all”. Metabolites were assigned KEGG classifications using the omu R package [41].

### Data analysis

R 4.4.2 [42] was used for all subsequent analyses. Univariate analyses were performed using Kruskal–Wallis tests followed by Dunn’s test [43] without *p*-value adjustment because of small sample size in each group [44].

Differences in the composition of species and lignocellulase gene families between treatments were assessed using nonmetric multidimensional scaling (NMDS; metaMDS function from vegan[45]) and Permutational MANOVA (*Perm*MANOVA) with 60,000 permutations (adonis2 function from vegan, after determining that between-group dispersion was not significantly different using the betadisper function with type = “median”) [45–47]. The envfit function was used to visualise how community composition related to standardised predictors with (significance assessed using stepwise deletion).

Indicator metabolites, taxa, and CAZy families for each treatment, and for 10-year bare *versus* both grassland treatments were assessed using the indval function from the labdsv package for R [46, 47] where indicator species were those with p values smaller than 0.05.

## Results

### Changes in soil carbon composition in response to the removal of plant C inputs

**Fig. 1:**
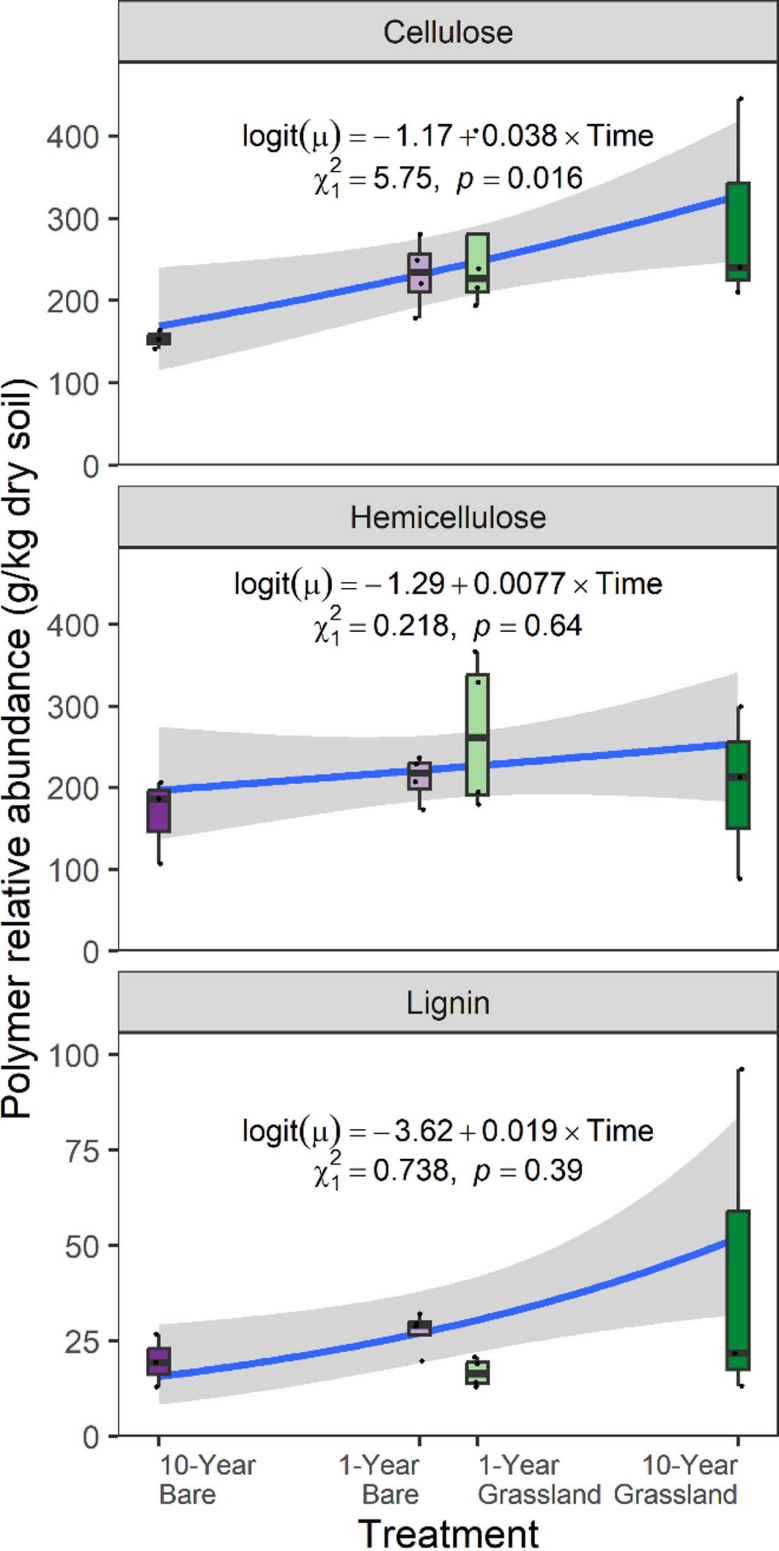
The relative abundance of lignocellulose polymers in the soil of grassland and plant-excluded plots in this experiment. The experimental treatments consisted of plant-excluded (bare) soil, and annually mown grasslands which had been established with these treatments for differing lengths of time. The treatments had sample sizes: 10-year bare N = 3, 1-year bare N = 4, 1-year grassland N = 4, 10-year grassland N = 3. The fitted lines represent generalized linear models (GLMs) with betabinomial error distributions which were fitted on proportional abundance data with being transformed back to g/kg dry soil. The x-axis represents time since establishment of the treatment with time expressed linearly from the centre of the axis (i.e. − 10 to 10); this was the predictor used in the GLM. Results shown in the figure are from a likelihood-ratio test on the fitted GLM

Changes in soil properties were presented in [15]George et al*.* (2021) [15], demonstrating differences in total C between grassland and bare treatments, with significant reduction in 10-year bare plots (2.54%, SD = 0.58%) relative to the two grassland treatments (10-year grassland: 3.73%, SD = 0.3%, 1-year grassland: 3.57%, SD = 0.37%). For the 1-year bare soil though mean C content was reduced compared to both grassland treatments, this was not yet significant (3.11%, SD = 0.32%). Analyses of soil carbohydrates revealed cellulose was similarly reduced by long-term plant removal (Kruskal–Wallis test: χ^2^_3_ = 6.843, *p* = 0.077; 10-year bare: 15.29%, SD = 1.12%; 1-year bare: 23.22%, SD = 4.30%; 1-year grassland: 26.35, SD = 9.68%; 10-year grassland: 29.86%, SD = 12.79%; Fig. 1). The proportion of hemicellulose and lignin was however unaffected by experimental treatment (hemicellulose: Kruskal–Wallis test: χ23 = 2.138, *p* = 0.542; lignin: Kruskal–Wallis test: χ23 = 5.091, *p* = 0.165; Fig. 1).

### Impact of plant-derived C inputs on the soil microbial community

**Fig. 2:**
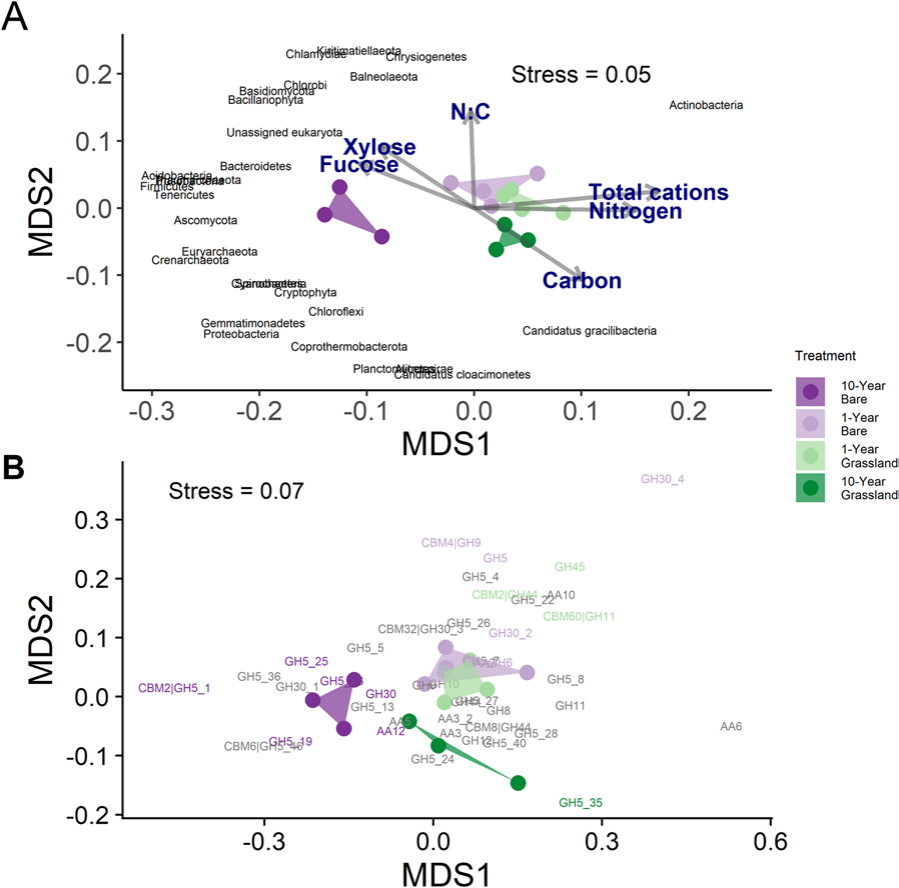
Nonmetric multidimensional scaling (NMDS) ordinations showing the effect of excluding plants inputs on **A** microbial community composition (PermMANOVA: F_3,10_ = 2.75, *p* < 0.001) and **B** lignocellulase gene composition (PermMANOVA: F_3, 10_ = 3.84, *p* < 0.001) as measured by metagenomics, showing correlations with soil chemistry. Arrows show the direction which is maximally correlated with environmental parameters. Weighted average scores of carbohydrate-active enzyme (CAZy) gene families are shown, and phyla are mapped on using the envfit function from vegan. CAZy families are coloured according to the treatment that they are indicator gene families for, with grey representing no treatment association. The experimental treatments consisted of plant-excluded (bare) soil, and annually mown grasslands which had been established with these treatments for differing lengths of time. The treatments had sample sizes: 10-year bare N = 3, 1-year bare N = 4, 1-year grassland N = 4, 10-year grassland N = 3

Analyses of taxonomic composition based on numbers of sequencing reads mapping to all assembled contigs revealed community composition at the species level was significantly impacted by experimental treatment (*Perm*MANOVA: F_3,10_ = 2.75, *p* < 0.001; Fig. 2A), with ordinations revealing highly distinct communities in the 10-year bare treatment. At the phylum level, Bacteroidetes, Acidobacteria, Firmicutes, Fusobacteria, Tenericutes, Ascomycota, Thaumarchaeota, Euryarchaeota, Crenarchaeota and others were associated with the 10-year bare plots following grouping of taxa and linear fitting of response vectors to the ordination (Fig. 2A). Further analyses of taxon level responses (genus or highest obtained classification) are presented in Fig. S3 and revealed phylogenetic signal in the response to carbon deprivation.

### Lignocellulolytic potential of the microbial community

**Fig. 3:**
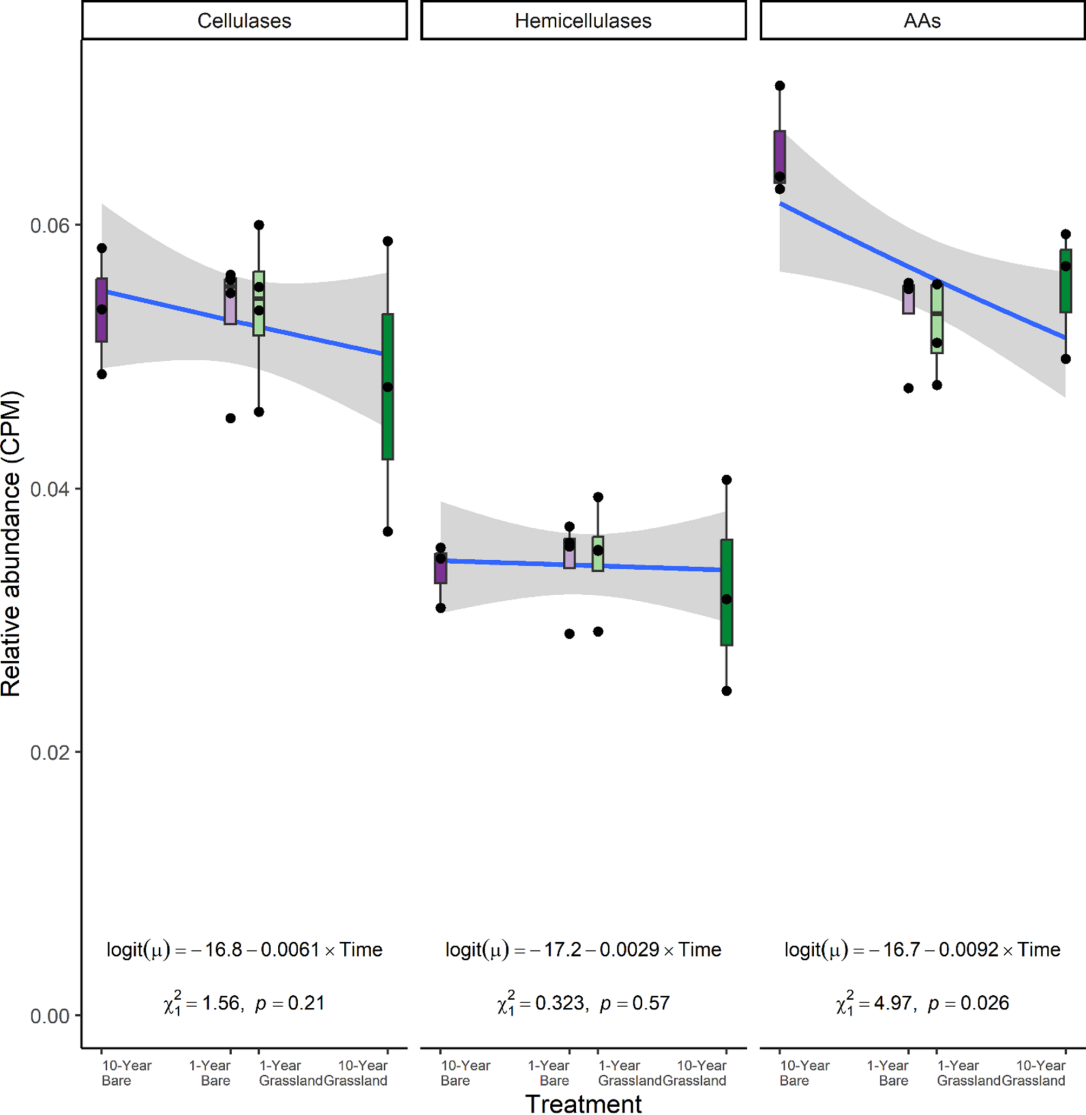
The relative abundance of different lignocellulolytic carbohydrate-active enzyme (CAZy) gene classes in the soil of grassland and plant-excluded (bare) plots in this experiment, according to metagenomes. The experimental treatments consisted of plant-excluded (bare) soil, and annually mown grasslands which had been established with these treatments for differing lengths of time. The treatments had sample sizes: 10-year bare N = 3, 1-year bare N = 4, 1-year grassland N = 4, 10-year grassland N = 3. The fitted lines represent generalized linear models with beta-binomial error distributions which were fitted on proportional gene abundance data before being transformed back to length-scaled counts per million (CPM; analogous to transcripts per million when data are not transcripts). The x-axes represent time since establishment of the treatment, with time expressed linearly from the centre of each axis (i.e. − 10 to 10); this was the predictor used in the generalized linear model (GLM) and is represented as “Time” in the equations of the lines drawn. Error bars around the fitted line represent 1 standard error. Results shown in the figure are from a likelihood-ratio test on the fitted GLM

Assessment of lignocellulose degrading genes was carried out following mapping of sequence reads to all contigs containing an identified CAZyme gene. Multivariate analyses of CAZyme abundance profiles revealed similar results to the taxonomic assessment with distinct clustering of the 10-year bare plots (Fig. 2B; *Perm*MANOVA: F_3, 10_ = 3.84, *p* < 0.001). Again, whilst the 1-year bare plots appeared to be distinct from the 10-year grasslands there were only minor differences from the 1-year grassland plots, meaning there were only limited direct effects of plant removal in the short-term on the CAZyme profiles. When relative abundances of all CAZymes for the three functional classes were summed there were no differences in the abundances of total lignocellulase genes, cellulase genes or xylanase genes (Kruskal–Wallis tests all with 3 d.f, *p* > 0.05), however, auxiliary activity genes were more abundant in the 10-year bare treatment than in both 1-year and grassland soils (Fig. 3; Kruskal–Wallis test: Χ^2^_3_ = 7.54, *p* = 0.056), likely because of elevated abundances of specific AAs noted above (Fig. 2B). We observed reductions to the richness of cellulase, xylanase and AA gene families under both 10-year treatments (Figs. S4–S6). An indicator species analysis was conducted to identify genes specifically associated with 10-year bare plots (Fig. S7). This revealed 7 indicators of long-term bare treatments, including three auxiliary activity genes, commonly implicated in lignin degradation. Notably, no auxiliary activity genes were significantly enriched in grassland or 1-year bare soils. The most significant indicator of bare soils was AA12, and this was notably the only AA indicator in this study. Three cellulose-degrading CAZy subfamilies from the GH5 family were also elevated in 10-year bare, though we were unable to functionally distinguish these from three other GH5 subfamilies which were indicators of grassland soils. Similarly, specific putative xylanase subfamilies from the GH30 family were both indicators of bare and grassland soils.

### Linkages between taxonomic and functional indicators of long-term bare soil

**Fig. 4:**
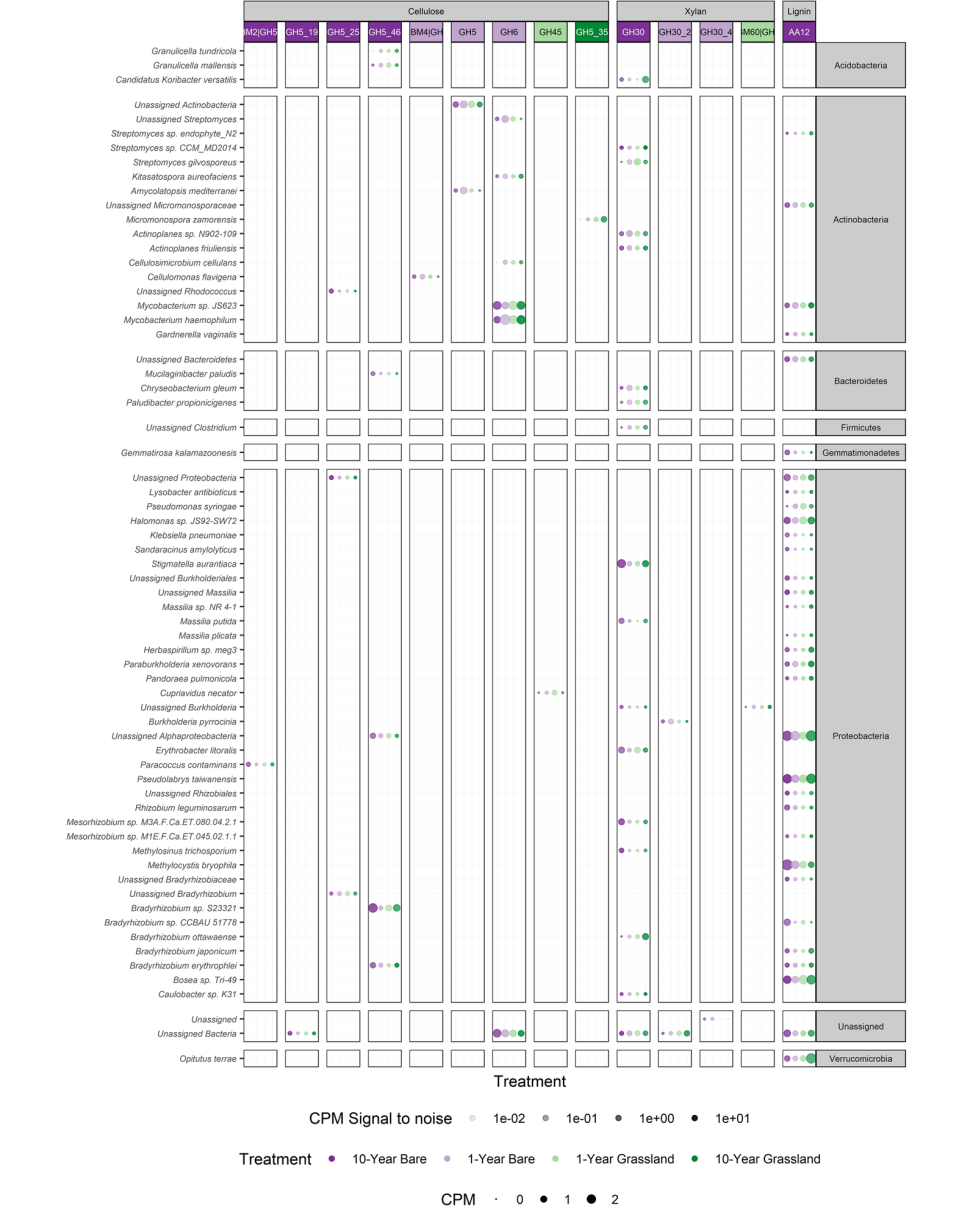
Relative abundances (in counts per million, CPM) and taxonomic origins of indicator lignocellulase carbohydrate-active enzyme (CAZy) gene families of contigs generated from metagenomic sequencing in this study that could be taxonomically assigned. The experimental treatments consisted of plant-excluded (bare) soil, and annually mown grasslands which had been established with these treatments for differing lengths of time. The plot shows species-level taxonomic origins of indicator lignocellulolytic gene families. Relative abundances are represented by point size. The treatments had sample sizes: 10-year bare N = 3, 1-year bare N = 4, 1-year grassland N = 4, 10-year grassland N = 3. CPM is the same as transcripts per million when data are not transcripts

To link changes in function with taxonomy, we analysed the taxonomic origins of the lignocellulase genes that were associated with different treatments (Figs. 4, S7). The six indicator lignocellulolytic CAZy (sub)families of the 10-year bare plots came from 55 taxonomically resolved groups, highlighting the relatively small diversity of microbial species which are likely most involved in lignocellulose degradation in soils (Fig. 4).

The 10-year bare indicator cellulase and xylanase families (Figs. 2B, S7) had sparse taxonomic origins (Fig. 4), with some of these genes being associated with indicator species. The 10-year bare indicator ligninase families were mostly associated with *Proteobacteria* although *Actinobacteria* and other phyla also possessed these genes (Fig. 4). The indicator cellulase families for the 1-year bare treatment (GH5, GH6, CBM4|GH9) had origins exclusively from across *Actinobacteria* or contigs which could not be assigned at the phylum level. When the indicator analysis was performed on the 10-year bare soils *versus* both grassland treatments, the only significant indicator gene families were GH5 and GH5_25, both of which served as indicators of the long-term bare soils, and which were associated with the unknown *Bradyrhizobium* indicator species.

### Short- and long-term plant removal dramatically affects soil metabolomes

**Fig. 5:**
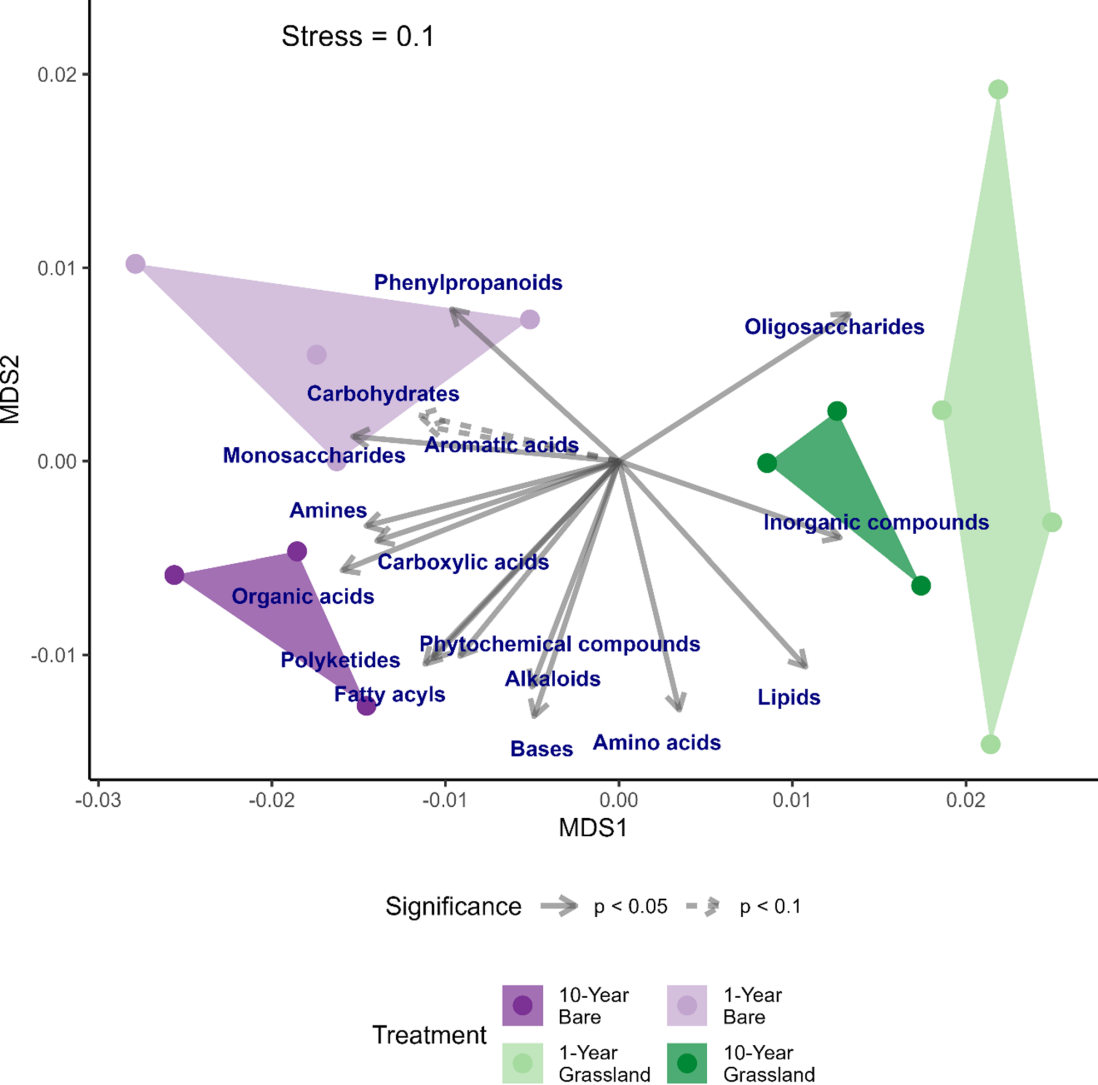
A nonmetric multidimensional scaling (NMDS) ordination of soil metabolite composition, showing the effect of plant-exclusion in grassland soils which are either mown annually or have had plants removed (bare; data PermMANOVA: F_3,10_ = 4.65, *p* = 0.003). Arrows and text show the direction which is maximally correlated with KEGG Level 2 classifications

Ordination of metabolome profiles revealed much clearer separation of both 10- and 1-year bare soils from the vegetated soils compared with the metagenomics data (Fig. 5; *Perm*MANOVA: F_3,10_ = 4.65, *p* = 0.003). Bare soils were significantly enriched in many extractable metabolites compared to vegetated soils. An indicator species analysis comparing 10- and 1-year bare versus all grass samples revealed significant enrichment of 135 metabolites in bare versus only 39 in grassland. Of the identified metabolites, oligosaccharides were key indicators of vegetated soils (trehalose, raffinose, isomaltose) alongside specific monosaccharides (1-kestose, myo-inositol, UDP-N-acetylglucosamine, galactinol, 1-kestose), lipids (palmitoleic acid, glycerol-alpha-phosphate, glycerol-3-galactoside), phospholipid headgroup components (phosphoethanolamine), and amino acids (tyrosine, valine, isoleucine, glutamine). Additionally, secondary metabolites such as beta-sitosterol and 4′,5-dihydroxy-7-glucosyloxyflavanone signify plant-derived inputs in vegetated soils. In bare soils, there was a greater prevalence of diverse compounds including pentose monosaccharides (xylose, xylulose, ribose, lyxose), hexoses and sugar alcohols/acids (fucose, 6-deoxyglucose, erythrose, erythritol, xylitol, glyceric acid); pyrimidine nucleotide bases (thymine, cytosin); amines (tyramine); amino acids (ornithine, methionine, glycocyamine); carboxylic acids (pyruvic, lactic, 3-hydroxybutyric, 4-hydroxybutyric, and 4-hydroxybenzoic acids). Additionally, fatty acids (e.g., pimelic, palmitic,myristic, capric, 5-aminovaleric and pentadecanoic acids), long-chain alcohols (e.g., dodecanol), isothreonic acid, the vitamin pantothenic acid, and other acids were significantly enriched in bare soils.

## Discussion

### Long-term bare soils show evidence of energy limitation

Our study demonstrates that the removal of plant carbon (C) inputs significantly alters soil chemical properties, microbial communities and their functional potential with respect to plant material degradation, as measured through metagenomics. Substantial reductions in soil C content in long-term bare soils [15] were associated with reduced cellulose content, but hemicellulose and lignin contents were unaffected (Fig. 1), suggesting a reliance of the microbial community on cellulose as a primary C source. In the 1-year bare plots, though SOM appeared to be decreasing, there were no differences in relative abundances of any of the lignocellulose component contents. We expected to see a decrease in the relative proportions of hemicellulose and lignin alongside the cellulose in the 10-year bare plots, as decomposable matter is utilised and as more plant matter is added to the soil in the grassland treatments. We saw no change in hemicellulose and lignin content between treatments, despite abundant indicator metabolites associated with hemicellulose and lignin breakdown in bare plots (*e.g.* xylose, mannose, fucose, vanillic acid, 4-hydroxybenzoic acid, ferulic acid, Fig. 5; Additional file 10). Because of this, we posit that the readily degradable fractions of hemicellulose and lignin are quickly degraded, or in the case of lignin, modified (likely to access cellulose and thus cellobiose) [48], whilst leaving less modifiable structures intact. The low abundance of lignin relative to cellulose in grassland species may also be a factor driving these results as changes become harder to detect. Future work on such systems could employ methods which quantify and distinguish lignin monomer types, to investigate differences in microbial degradation selectivity. Because of the long-term nature of this experiment and the limited number of experimental plots, our study is constrained by small sample size and therefore limited statistical power. This increases the chance of Type II errors (reducing our ability to detect potential treatment effects) and increases uncertainty around the effect size estimates. We also recognise that performing many tests inflates the chance of Type I errors (false positives). Given the need to integrate results from many complementary methods to understand changes to a complex soil system, p-value corrections were not applied, as these further increase the likelihood of false negatives. Given the exploratory nature of the work, we interpret our results cautiously and provide them as hypothesis generating, providing a foundation for future more highly replicated studies investigating the microbial basis of soil carbon cycling. 

### Long-term bare soils have altered microbial communities

Long-term exclusion of plants substantially altered soil microbial community composition, favouring many discrete taxa in 10-year bare soils, alongside reductions in fungal and bacterial biomass and richness previously reported [15]. We detected phylogenetic signal in the taxa which increased and decreased due to C deprivation, suggesting that conserved functional traits within clades shape the broader taxonomic composition of the microbial community (Additional file 3). Our data suggest that broadly distributed members of Firmicutes, Proteobacteria, Acidobacteria, Ascomycetes and Thaumarchaeota are the taxa most able to exploit remaining available soil C and nutrients in the context of plant exclusion (Fig. 2). Alternatively, taxa may rely on alternative metabolic strategies such as autotrophy [49]. In line with theory outlined by the C-S-R and Y-A-S life-history classification systems [50, 51], we initially expected that decreased availability of soil carbon, energy-rich cellulose, and plant exudates should favour ruderal, stress-tolerant, and resource acquisition-focused microorganisms [50, 51]. Malik et al. [51] in both systems, the reduced availability of preferred substrates [48] should limit the abundance of competitive, high-yielding species. The shift in beta diversity space from the 10-year bare treatment, outside of the area occupied by both grassland treatments (Fig. 2A), demonstrates that the dominant community members from the grassland plots are no longer dominant in the 10-year bare plots. Dominant community members in energy-rich, stable environments typically produce biomass rapidly or suppress competitors through resource monopolisation or interference competition [52, 53]. Our data suggest a shift toward species better adapted to acquiring and utilising resources in low-energy, resource-depleted conditions [54]. Indeed, the reduced GC content of the DNA from the 10-year bare soils (Additional file 8) is consistent with the microbial energy conservation and reduced nitrogen utilisation strategies associated with low pH soils [55]. One example lies in the increases in Thaumarchaeota which, due to their chemoautotrophic life-history [49], may be released from competition for nutrients, and particularly space [56]. Interestingly, our data for Thaumarchaeota reflects trends for this phylum in response to tillage [57] and agricultural management [58, 59], both of which are associated with reductions in soil carbon, and consequently energy.

### Lignin degradation potential increases under long-term plant exclusion

The 10-year bare soils had a strikingly increased relative abundance of AA genes. Whilst these are typically considered to be involved in lignin degradation, we did not detect differences in lignin content. It is possible that after only 10 years, the organisms expressing these genes have not yet significantly impacted the low amounts of lignin in the grassland system. Notably, Barré et al. (2018) [12] saw complete removal of lignin-based compounds after fifty years of bare-fallow. Later reassessment of these plots will therefore provide an opportunity to understand how generalisable complete utilisation of lignin is under bare conditions. One limitation of the lignocellulose analysis used is the inability to measure chemical differences in lignin composition. Significant modification of the lignin structures in bare plots may have been performed by brown-rot fungi and microorganisms that produce hydroxyl radicals [60] but we are unable to detect such changes with the available data. Alternatively, these AA genes may provide crucial activities on other substrates, as they have been implicated in the degradation of chitin, hemicellulose, and other complex polysaccharides, reflecting their versatile roles in enabling microbial metabolism [61–64]. The only AA indicator in this study, AA12, is classified as an oxidoreductase with activity screening results showing activity on both pentose and hexose monosaccharides, and in particular rare sugars [64, 65], possibly indicating general utilisation of scarce simple sugars under bare conditions. The summed abundances of cellulases and xylanases were unaffected by treatments (Fig. 3). We did not observe enrichment of cellulolytic gene families [66, 67] in the 10-year bare soils as predicted (Fig. 3), and richness was not clearly affected (Fig. S4). However, the decreased cellulose content in bare soil indicates that cellulolysis is an important C source for microorganisms in unvegetated soils, and we observed shifts in the composition of cellulase genes between treatments. It should be noted that other cellulolytic gene families were significant indicators of vegetated soils, and we were unable to ascribe any biochemical differences in putative substrates in the literature. Both 10-year treatments had reduced lignocellulolytic gene family richness (Figs. S4–S6) in each of the three broad functional classes. This may be due to ecosystem stability allowing effective ecological filtering of less well-adapted species to the dominant resource availabilities under each management class.

### Lignocellulase genes may offer competitive advantages for microorganisms in energy-limited soils

Because of the sparse genetic origins of these functional gene classes relative to the diversity of the community (3.2% of assigned taxonomic classifications had a lignocellulase gene, Fig. 4), changes to individual taxa hold the potential to significantly affect carbon cycling processes in grassland soils.

Indicator genes with more than one taxonomic origin were often coupled with several taxonomic increases, suggesting that the indicator gene families are beneficial for survival and reproduction under different conditions. For instance, differences in the long-term bare indicator GH5_25 were driven by increases of *Rhodococcus spp.* and contigs assigned as an unassigned proteobacterium (Figs. 4, S7). GH5_25 is often associated with multi-substrate specificity such as for β-mannan-based and β-glucan-based polymers [68], likely conferring advantages to species under resource deprivation by reducing the genetic load required to hydrolyse diverse substrates. Increases in this gene family therefore likely signify shifts towards generalist lignocellulose degraders. Another mechanism which could enable proliferation of many species is the decline in dominant utilisers of some resources under bare conditions; decreases to *Bradyrhizobium ottawaense* and the *Candidatus Koribacter versatilis* under bare conditions may have been a cause of GH30 genes being indicative of bare soils. Extra credence is given to this idea through previous findings of *Candidatus K. versatilis* as a potential degrader of hemicellulose side chains and cellulose [69]. The most common indicator, AA12, had ten times the number of taxonomic origins on average compared to other indicator gene families. Differences in this family were driven by increases in alphaproteobacterial sequences and decreases in betaproteobacterial sequences.

### Bare soils host abundant sugars and metabolites from plant tissue breakdown

Analyses of soil metabolites revealed the strongest effects of vegetation removal, with large differences even between the 1-year treatments. Concurring with the CAZyme analyses, we saw greater abundances of lignin breakdown products in both 1- and 10-year bare soils (*e.g.* vanillic acid, 4-hydroxybenzoic acid, benzoic acid). More surprising, this was accompanied by enrichment of a plethora of metabolites, likely arising from both plant breakdown and microbial metabolism. Interestingly, there was an association of monosaccharides, organic acids and fermentation products within the bare treatments, contrasted with an association of oligosaccharides, lipids, and inorganic compounds with the grassland treatments (Fig. 5). The accumulation of such compounds fits with recent theories on the persistence of organic matter being within small simple molecules. We can only speculate here on the likely mechanisms for this—why in energy-limited soils (reduced carbon and nitrogen content with reduced microbial biomass) do many sugars seem to persist? Simple answers such as N limitation may be invoked, but we note earlier work revealed an abundance of N in 1-year bare soils [15]. Similarly, it is possible that the bare soils are more anaerobic, due to reductions in soil structure [15, 70]. Indeed, alternative fermentative pathways were elevated in bare soil metagenomes (*e.g.* Lysine fermentation to acetate and butyrate, acetylene degradation, and succinate fermentation to butyrate), but conversely, more standard fermentation pathways (homo-heterolactic) were elevated in vegetated metagenomes. Other accessibility mechanisms may also be at play [12, 71–74], though it is thought that sugars, being uncharged, do not strongly bind soil particles and are at least somewhat available to microorganisms depending on soil type [75, 76]. More likely is that these breakdown products, particularly pentoses, are not expected to be favoured by microorganisms, especially under conditions of energy limitation where pentose metabolism is energetically costly and generally used for growth [77] and secondary metabolism [78]. Both this and microbial substrate preference for cellobiose (which may be at concentrations high enough for many soil microorganisms due to ongoing investment in cellulolysis; Figs. 2, 3B, 4) may provide an explanation for the accumulation of monosaccharides in bare soils in this study [48], and warrant further examination. The clear trends highlighted by the metabolomics data relative to those seen in the metagenomes show the importance of using techniques which access biological information in a timeframe that is as close to real-time as possible. Future work combining metagenomics with quantitative PCR, metatranscriptomics, and direct enzyme assays would more fully resolve the relationship between gene abundance, expression, and lignocellulose-degrading activity.

## Conclusion

Our data show that long-term (10-year) plant removal strongly affects soil microbial biodiversity and function. Bare soils exhibited signs of energy limitation (*i.e*., reduced C, N, microbial biomass) and reliance of microorganisms on plant biomass degradation, with elevated simple substrates in both short- and long-term bare plots. Future research should explore why lignin degradation increases despite steady abundance, the roles of CAZy genes that increase, and the rise in monosaccharide levels under plant exclusion. Advancing soil C cycling knowledge requires deeper insights into specific CAZy gene activities, taxonomic links, and the products of extracellular enzyme activity—especially in energy- and nutrient-limited systems. Extracellular enzymes may be the latch opening the door to the soil carbon cycling party, but it is likely the environment, and interactions with resident microbes and their specific metabolic activities, which determine the chemicals that persist.

## Supplementary Information

Below is the link to the electronic supplementary material.

Supplementary Material 1.

## Data Availability

The sequencing data generated in this study are accessible at the European Nucleotide Archive under the project accession PRJEB49281. Metabolomic trace files are deposited in the MetaboLights data repository under the accession MTBLS12728. Code and data for the analysis are available at https://github.com/fidlerdb/Plant_exclusion_experiment_lignocellulase_genes.
