# Supplementary material for: Soil microbial adaptation to carbon deprivation: shifts in lignocellulolytic gene profiles following long-term plant exclusion
Source: Environ Microbiome. 2025 Dec 10;21:9. doi: 10.1186/s40793-025-00810-6 (PMC12802329; doi:10.1186/s40793-025-00810-6)
Supplement: Supplementary file 1 — Supplementary Material 1. [file 40793_2025_810_MOESM1_ESM.docx]

# Additional files

**Additional file 1**


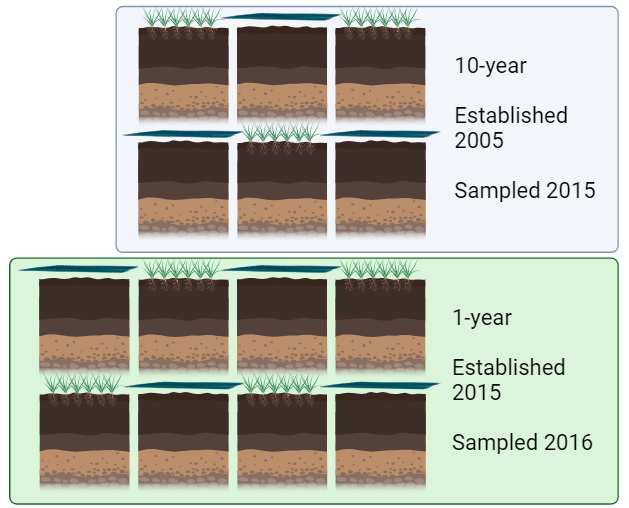


Schematic showing the experimental setup. Plots with grass in each of the age classes are “grassland” or “vegetated” plots, and samples without plants are “bare” or “unvegetated” or “plant-excluded” plots. The shape above each bare plot represents the permeable fabric kept over the bare plots to prevent desiccation and colonisation effects.

**Additional file 2**


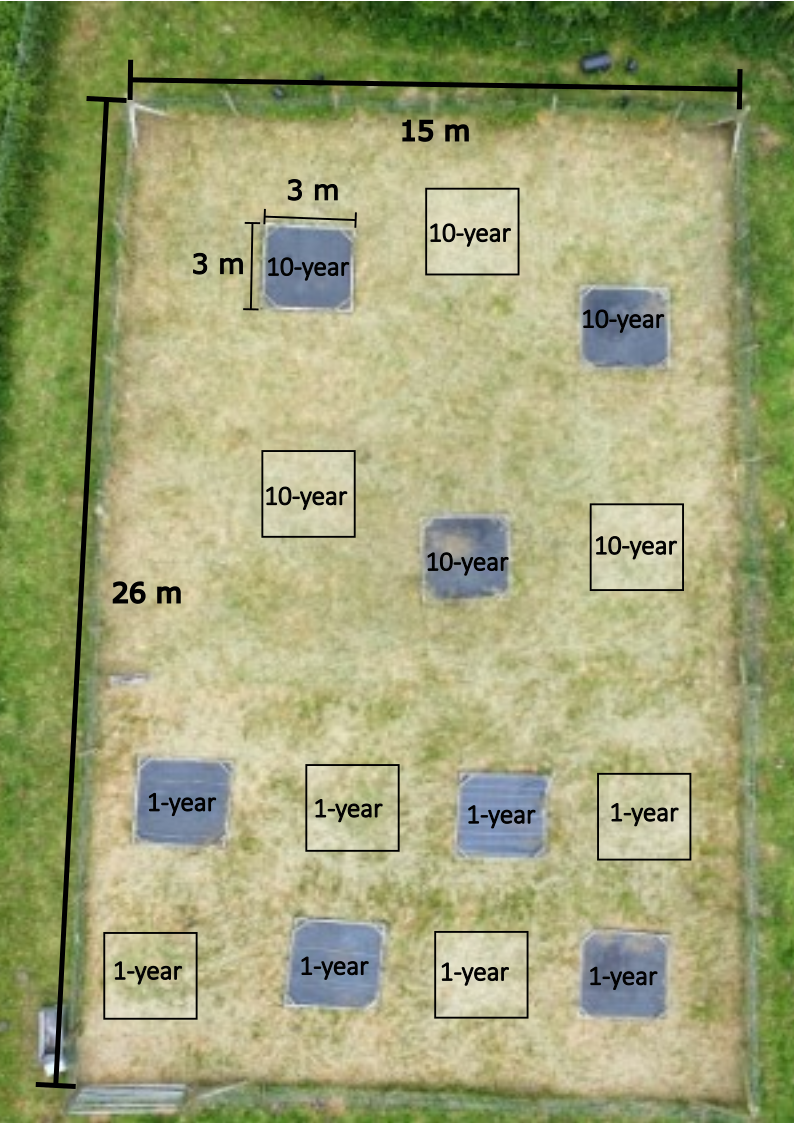


Aerial photograph of the experiment after annual mowing. The bare plots under black sheeting are clearly visible. Plots are marked by their age category. The experimental treatments consisted of plant-excluded (bare) soil, and annually mown grasslands which had been established with these treatments for differing lengths of time. The treatments had sample sizes: 10-year bare N=3, 1-year bare N=4, 1-year grassland N=4, 10-year grassland N=3.

**Additional file 3**


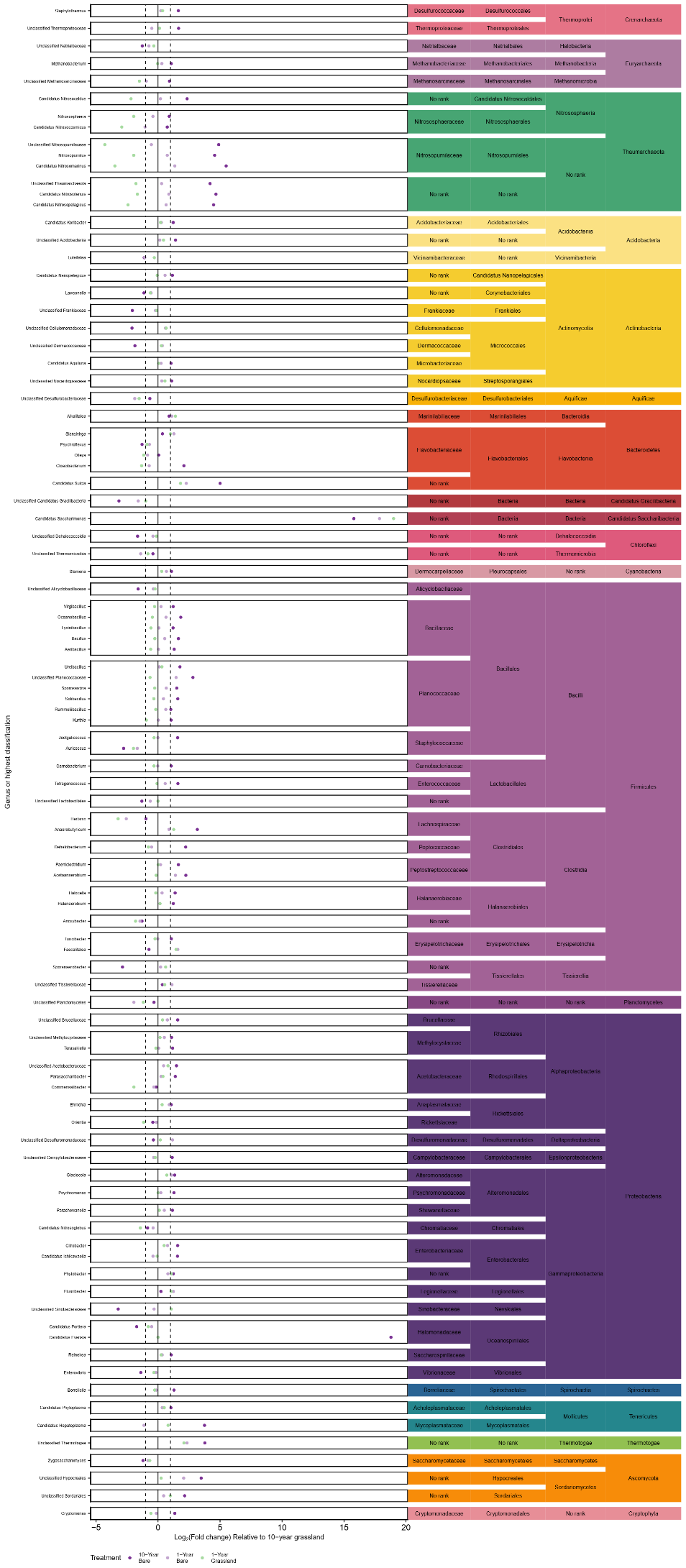


Changes in relative abundances of the species (grouped phylogenetically) which showed a significant log_2_ fold change relative to in 10-year grassland, according to GLMs. The experimental treatments consisted of plant-excluded (bare) soil, and annually mown grasslands which had been established with these treatments for differing lengths of time. The treatments had sample sizes: 10-year bare N=3, 1-year bare N=4, 1-year grassland N=4, 10-year grassland N=3.

**Additional file 4**


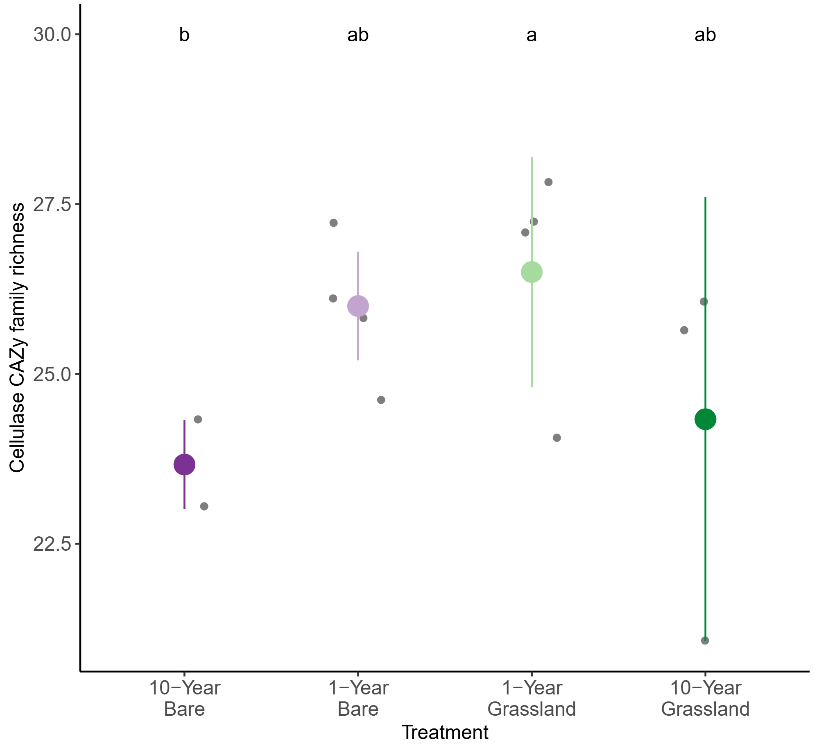


Richness of cellulase CAZy families in each treatment. Letters above the data represent significance groupings determined by a Dunn’s test, where groups which share a letter were not significantly different. The experimental treatments consisted of plant-excluded (bare) soil, and annually mown grasslands which had been established with these treatments for differing lengths of time. The treatments had sample sizes: 10-year bare N=3, 1-year bare N=4, 1-year grassland N=4, 10-year grassland N=3. Errorbars represent Gaussian 95% confidence intervals.

**Additional file 5**


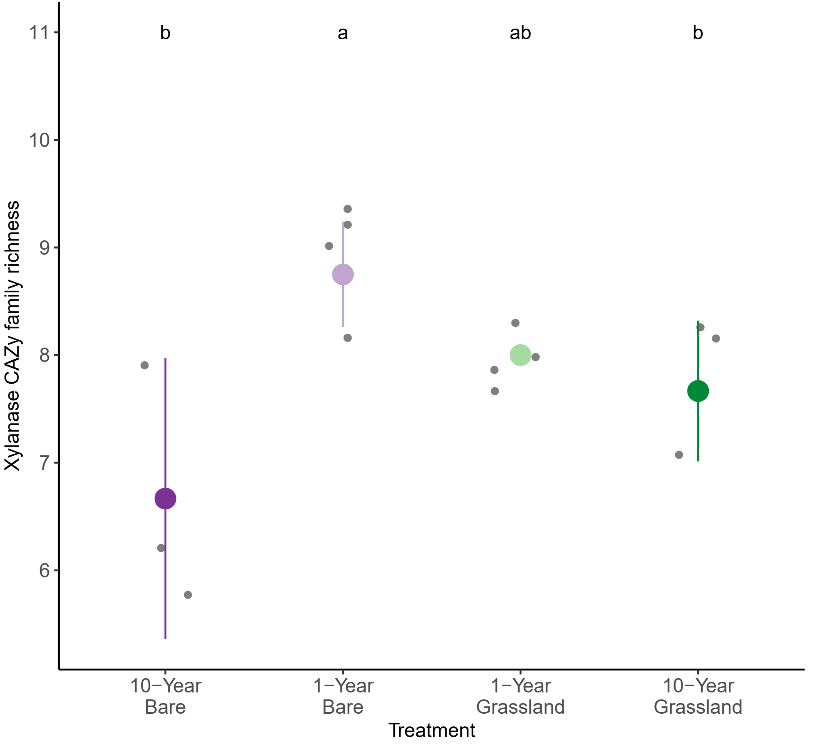


Richness of xylanase CAZy families in each treatment. Letters above the data represent significance groupings determined by a Dunn’s test, where groups which share a letter were not significantly different. The experimental treatments consisted of plant-excluded (bare) soil, and annually mown grasslands which had been established with these treatments for differing lengths of time. The treatments had sample sizes: 10-year bare N=3, 1-year bare N=4, 1-year grassland N=4, 10-year grassland N=3. Errorbars represent Gaussian 95% confidence intervals.

**Additional file 6**

https://github.com/fidlerdb/Plant_exclusion_experiment_lignocellulase_genes/tree/main/Supplementary_figures/Species_changes_2022-05-10_supplementary.pdf


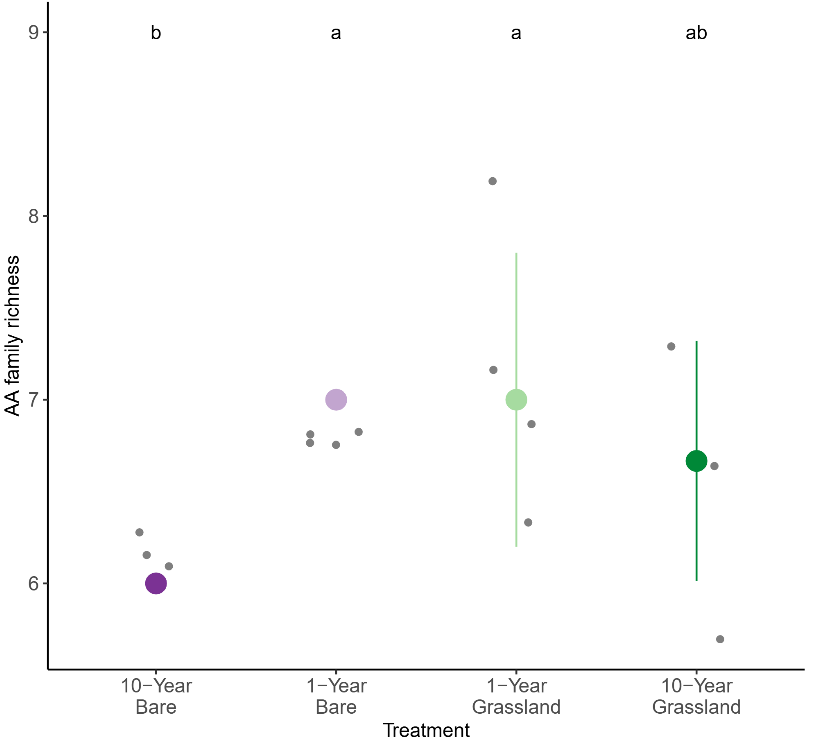


Richness of AA CAZy families in each treatment. Letters above the data represent significance groupings determined by a Dunn’s test, where groups which share a letter were not significantly different. The experimental treatments consisted of plant-excluded (bare) soil, and annually mown grasslands which had been established with these treatments for differing lengths of time. The treatments had sample sizes: 10-year bare N=3, 1-year bare N=4, 1-year grassland N=4, 10-year grassland N=3. Errorbars represent Gaussian 95% confidence intervals.

**Additional file 7**


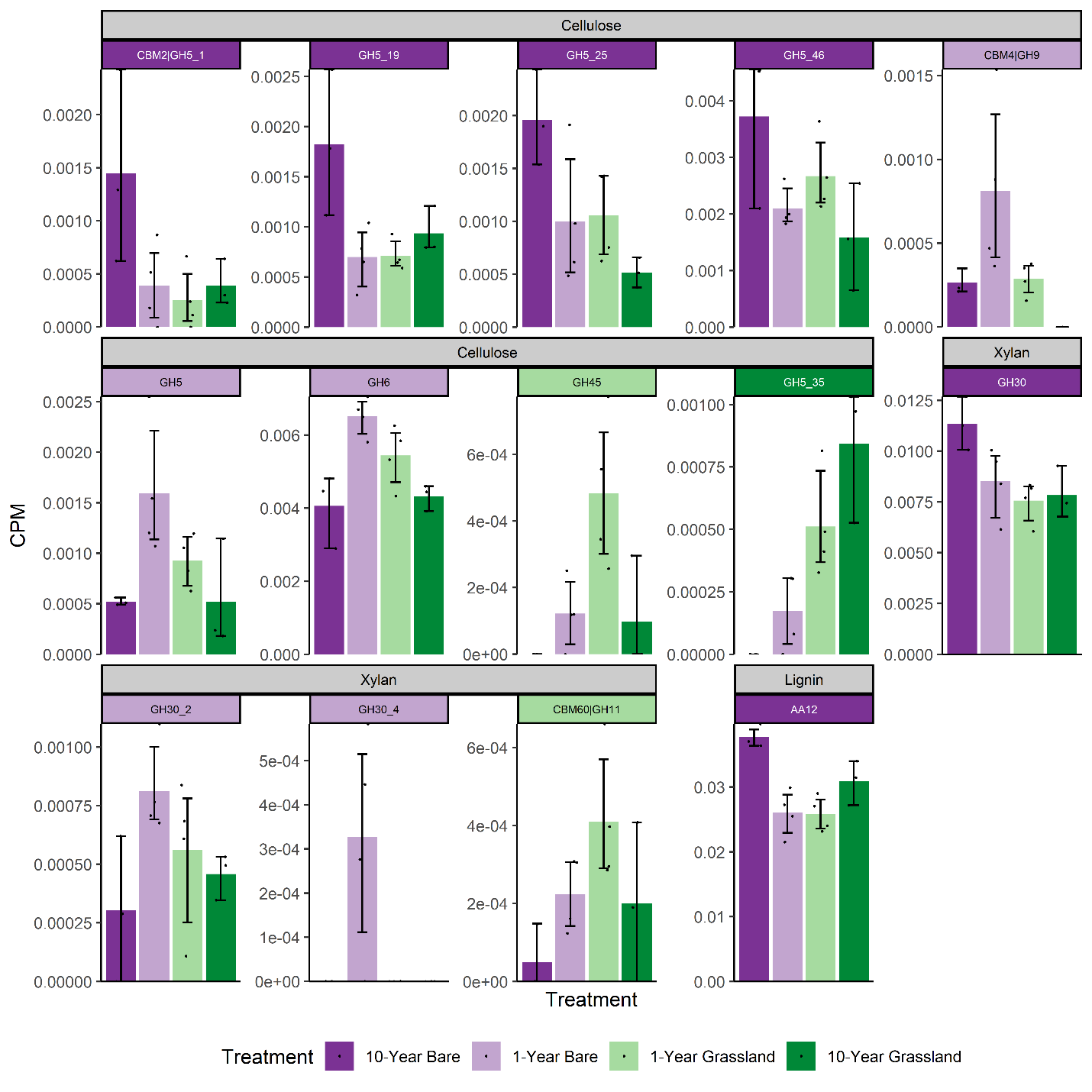


Relative abundances of indicator lignocellulase gene families of contigs generated from metagenomic sequencing in this study that could be taxonomically assigned. The experimental treatments consisted of plant-excluded (bare) soil, and annually mown grasslands which had been established with these treatments for differing lengths of time. The treatments had sample sizes: 10-year bare N=3, 1-year bare N=4, 1-year grassland N=4, 10-year grassland N=3.

**Additional file 8**

Read numbers and quality scores per metagenomic library from the Illumina HiSeq 4000 run.

| Sample | Total number of reads (pre-quality control) | Total number of reads (post-quality control) | Number of unpaired reads | Mean phred score ± SD | | Data volume (Gbp) | G+C content (%) | |
| --- | --- | --- | --- | --- | --- | --- | --- | --- |
|  |  |  |  | Forward read | Reverse read | Paired, Unpaired | Forward read | Reverse read |
| c1 | 25599822 | 25026515 | 98591 | 39.0 ± 4.2 | 38.9 ± 4.6 | 0.573, 0.002 | 62 | 62 |
| c2 | 40423536 | 40058096 | 308146 | 39.0 ± 4.3 | 38.3 ± 5.5 | 0.914, 0.000 | 62 | 62 |
| c3 | 39824734 | 39355430 | 356814 | 39.0 ± 4.3 | 38.1 ± 5.7 | 0.897, 0.008 | 63 | 63 |
| c4 | 44174930 | 43824259 | 306967 | 39.1 ± 4.2 | 38.3 ± 5.4 | 1.001, 0.007 | 63 | 63 |
| c5 | 41863660 | 41533356 | 260384 | 39.0 ± 4.3 | 38.4 ± 5.3 | 0.979, 0.006 | 63 | 63 |
| c6 | 37902940 | 37646088 | 213910 | 39.0 ± 4.2 | 38.5 ± 5.1 | 0.861, 0.005 | 63 | 63 |
| c7 | 34485136 | 33998556 | 290584 | 39.0 ± 4.3 | 38.0 ± 5.8 | 0.775, 0.007 | 63 | 62 |
| b1 | 34443578 | 34151212 | 248010 | 39.1 ± 4.2 | 38.3 ± 5.4 | 0.780, 0.006 | 61 | 61 |
| b2 | 39414296 | 39109814 | 280080 | 39.0 ± 4.3 | 38.2 ± 5.5 | 0.893, 0.006 | 62 | 62 |
| b3 | 45395230 | 44911778 | 462252 | 39.0 ± 4.3 | 38.0 ± 5.8 | 1.022, 0.011 | 62 | 62 |
| b4 | 39524656 | 39133275 | 331003 | 39.0 ± 4.2 | 38.2 ± 5.6 | 0.892, 0.008 | 63 | 63 |
| b5 | 38993776 | 38673647 | 274305 | 39.0 ± 5.2 | 38.3 ± 5.4 | 0.883, 0.006 | 63 | 63 |
| b6 | 43317956 | 42870965 | 303901 | 39.0 ± 4.3 | 38.3 ± 5.4 | 0.979, 0.007 | 63 | 63 |
| b7 | 37423404 | 37124607 | 239113 | 39.0 ± 4.2 | 38.4 ± 5.3 | 0.848, 0.005 | 62 | 62 |
| n1 | 338388 | 22095 | 409 | 39.2 ± 4.0 | 39.3 ± 4.1 | 0.000, 0.000 |  |  |

**Additional file 9**

Indicator analysis values for lignocellulase gene families.

| **CAZy family** | **Indicator** | **indval** | **pvalue** |
| --- | --- | --- | --- |
| AA12 | 10-Year Bare | 0.313101 | 0.008 |
| AA7 | NA | NA | NA |
| AA10 | NA | NA | NA |
| AA3 | NA | NA | NA |
| AA3_2 | NA | NA | NA |
| AA6 | NA | NA | NA |
| AA5 | NA | NA | NA |
| GH9 | NA | NA | NA |
| GH8 | NA | NA | NA |
| GH44 | NA | NA | NA |
| GH5_35 | 10-Year Grassland | 0.552273 | 0.013 |
| GH5_7 | NA | NA | NA |
| GH5_8 | NA | NA | NA |
| GH5_13 | NA | NA | NA |
| GH5_5 | NA | NA | NA |
| GH6 | 1-Year Bare | 0.320373 | 0.008 |
| GH5_46 | 10-Year Bare | 0.369725 | 0.045 |
| GH5_26 | NA | NA | NA |
| GH5 | 1-Year Bare | 0.44705 | 0.015 |
| GH5_24 | NA | NA | NA |
| GH5_40 | NA | NA | NA |
| GH5_19 | 10-Year Bare | 0.437493 | 0.017 |
| GH5_25 | 10-Year Bare | 0.43214 | 0.013 |
| GH5_4 | NA | NA | NA |
| GH5_28 | NA | NA | NA |
| GH5_27 | NA | NA | NA |
| GH12 | NA | NA | NA |
| GH5_36 | NA | NA | NA |
| GH45 | 1-Year Grassland | 0.685959 | 0.003 |
| CBM2.GH5_1 | 10-Year Bare | 0.582339 | 0.035 |
| GH5_22 | NA | NA | NA |
| CBM4.GH9 | 1-Year Bare | 0.59391 | 0.007 |
| CBM2.GH44 | 1-Year Grassland | 0.443864 | 0.042 |
| CBM8.GH44 | NA | NA | NA |
| CBM6.GH5_46 | NA | NA | NA |
| GH10 | NA | NA | NA |
| GH30 | 10-Year Bare | 0.321544 | 0.004 |
| GH11 | NA | NA | NA |
| GH30_2 | 1-Year Bare | 0.380881 | 0.045 |
| GH30_4 | 1-Year Bare | 0.75 | 0.024 |
| GH30_1 | NA | NA | NA |
| CBM32.GH30_3 | NA | NA | NA |
| CBM60.GH11 | 1-Year Grassland | 0.464068 | 0.047 |

**Additional file 10**

Indicator analysis values for metabolomics data.

| **Metabolite** | **Class** | **Subclass_1** | **Subclass_2** | **Treatment** | **indval** | **pvalue** |
| --- | --- | --- | --- | --- | --- | --- |
| 3,6-anhydro-D-galactose | Carbohydrates | Monosaccharides | Aldoses |  |  |  |
| erythrose | Carbohydrates | Monosaccharides | Aldoses | Bare | 0.664 | 0.001 |
| lyxose | Carbohydrates | Monosaccharides | Aldoses | Bare | 0.612 | 0.001 |
| mannose | Carbohydrates | Monosaccharides | Aldoses |  |  |  |
| ribose | Carbohydrates | Monosaccharides | Aldoses | Bare | 0.78 | 0.001 |
| threose | Carbohydrates | Monosaccharides | Aldoses |  |  |  |
| xylose | Carbohydrates | Monosaccharides | Aldoses | Bare | 0.687 | 0.001 |
| N-acetylmannosamine | Carbohydrates | Monosaccharides | Amino sugars |  |  |  |
| UDP-N-acetylglucosamine | Carbohydrates | Monosaccharides | Amino sugars | Grassland | 0.667 | 0.002 |
| n-acetyl-d-hexosamine | Carbohydrates | Monosaccharides | Amino sugars |  |  |  |
| fucose | Carbohydrates | Monosaccharides | Deoxy sugars | Bare | 0.711 | 0.003 |
| 1-kestose | Carbohydrates | Monosaccharides | Ketoses | Grassland | 0.75 | 0.004 |
| glucoheptulose | Carbohydrates | Monosaccharides | Ketoses |  |  |  |
| tagatose | Carbohydrates | Monosaccharides | Ketoses |  |  |  |
| xylulose | Carbohydrates | Monosaccharides | Ketoses | Bare | 0.717 | 0.003 |
| glyceric acid | Carbohydrates | Monosaccharides | Sugar acids | Bare | 0.581 | 0.031 |
| 1,5-anhydroglucitol | Carbohydrates | Monosaccharides | Sugar alcohols |  |  |  |
| erythritol | Carbohydrates | Monosaccharides | Sugar alcohols |  |  |  |
| galactinol | Carbohydrates | Monosaccharides | Sugar alcohols | Grassland | 0.582 | 0.036 |
| glycerol | Carbohydrates | Monosaccharides | Sugar alcohols |  |  |  |
| inositol-4-monophosphate | Carbohydrates | Monosaccharides | Sugar alcohols |  |  |  |
| lyxitol | Carbohydrates | Monosaccharides | Sugar alcohols |  |  |  |
| myo-inositol | Carbohydrates | Monosaccharides | Sugar alcohols | Grassland | 0.632 | 0.004 |
| pentitol | Carbohydrates | Monosaccharides | Sugar alcohols |  |  |  |
| ribitol | Carbohydrates | Monosaccharides | Sugar alcohols |  |  |  |
| threitol | Carbohydrates | Monosaccharides | Sugar alcohols |  |  |  |
| xylitol | Carbohydrates | Monosaccharides | Sugar alcohols | Bare | 0.7 | 0.002 |
| 6-deoxyglucose | Carbohydrates | Monosaccharides |  | Bare | 0.72 | 0.002 |
| fructose | Carbohydrates | Monosaccharides |  |  |  |  |
| glucose | Carbohydrates | Monosaccharides |  |  |  |  |
| glucose-1-phosphate | Carbohydrates | Monosaccharides |  |  |  |  |
| gluconic acid | Carbohydrates |  |  | Bare | 0.673 | 0.006 |
| isomaltose | Carbohydrates | Oligosaccharides | Disaccharides | Grassland | 0.636 | 0.001 |
| sophorose | Carbohydrates | Oligosaccharides | Disaccharides |  |  |  |
| sucrose | Carbohydrates | Oligosaccharides | Disaccharides |  |  |  |
| trehalose | Carbohydrates | Oligosaccharides | Disaccharides | Grassland | 0.576 | 0.007 |
| melezitose | Carbohydrates | Oligosaccharides | Trisaccharides |  |  |  |
| raffinose | Carbohydrates | Oligosaccharides | Trisaccharides | Grassland | 0.732 | 0.029 |
| phosphate | Inorganic compounds |  |  | Grassland | 0.785 | 0.001 |
| isothreonic acid | Lipids | Fatty acyls | Eicosanoids |  |  |  |
| 4-aminobutyric acid | Lipids | Fatty acyls | Fatty Acids and Conjugates |  |  |  |
| 5-aminovaleric acid | Lipids | Fatty acyls | Fatty Acids and Conjugates | Bare | 0.66 | 0.002 |
| arachidic acid | Lipids | Fatty acyls | Fatty Acids and Conjugates |  |  |  |
| arachidonic acid | Lipids | Fatty acyls | Fatty Acids and Conjugates |  |  |  |
| behenic acid | Lipids | Fatty acyls | Fatty Acids and Conjugates |  |  |  |
| capric acid | Lipids | Fatty acyls | Fatty Acids and Conjugates | Bare | 0.592 | 0.018 |
| glutaric acid | Lipids | Fatty acyls | Fatty Acids and Conjugates |  |  |  |
| lignoceric acid | Lipids | Fatty acyls | Fatty Acids and Conjugates |  |  |  |
| linoleic acid | Lipids | Fatty acyls | Fatty Acids and Conjugates |  |  |  |
| linolenic acid | Lipids | Fatty acyls | Fatty Acids and Conjugates |  |  |  |
| myristic acid | Lipids | Fatty acyls | Fatty Acids and Conjugates | Bare | 0.584 | 0.027 |
| nonadecanoic acid | Lipids | Fatty acyls | Fatty Acids and Conjugates |  |  |  |
| oleic acid | Lipids | Fatty acyls | Fatty Acids and Conjugates |  |  |  |
| palmitic acid | Lipids | Fatty acyls | Fatty Acids and Conjugates | Bare | 0.565 | 0.014 |
| palmitoleic acid | Lipids | Fatty acyls | Fatty Acids and Conjugates | Grassland | 0.685 | 0.015 |
| pentadecanoic acid | Lipids | Fatty acyls | Fatty Acids and Conjugates | Bare | 0.56 | 0.039 |
| pimelic acid | Lipids | Fatty acyls | Fatty Acids and Conjugates | Bare | 0.606 | 0.01 |
| 1-hexadecanol | Lipids | Fatty acyls | Fatty alcohols |  |  |  |
| dodecanol | Lipids | Fatty acyls | Fatty alcohols | Bare | 0.619 | 0.048 |
| 1-monopalmitin | Lipids | Glycerolipids | Monoradylglycerols |  |  |  |
| 1-monostearin | Lipids |  |  |  |  |  |
| glycerol-3-galactoside | Lipids |  |  | Grassland | 0.658 | 0.017 |
| glycerol-alpha-phosphate | Lipids |  |  | Grassland | 0.885 | 0.003 |
| daidzein | Lipids | Polyketides | Flavonoids | Bare | 0.643 | 0.002 |
| deoxycholic acid | Lipids | Sterol Lipids | Bile acids and derivatives |  |  |  |
| adenine | Nucleic acids | Bases | Purines |  |  |  |
| guanine | Nucleic acids | Bases | Purines |  |  |  |
| cytosin | Nucleic acids | Bases | Pyrimidines | Bare | 0.596 | 0.005 |
| thymine | Nucleic acids | Bases | Pyrimidines | Bare | 0.594 | 0.023 |
| uracil | Nucleic acids | Bases | Pyrimidines |  |  |  |
| urea | Nucleic acids |  |  |  |  |  |
| thymidine | Nucleic acids | Nucleosides | Deoxyribonucleosides |  |  |  |
| adenosine | Nucleic acids | Nucleosides | Ribonucleosides |  |  |  |
| vanillic acid | Organic acids | Aromatic acids | monocyclic |  |  |  |
| hydroquinone | Organic acids | Aromatic acids |  |  |  |  |
| 2-ketoadipic acid | Organic acids | Carboxylic acids | 2-Oxocarboxylic acids |  |  |  |
| 2-ketoisocaproic acid | Organic acids | Carboxylic acids | 2-Oxocarboxylic acids |  |  |  |
| pyruvic acid | Organic acids | Carboxylic acids | 2-Oxocarboxylic acids | Bare | 0.659 | 0.035 |
| adipic acid | Organic acids | Carboxylic acids | Dicarboxylic acids |  |  |  |
| fumaric acid | Organic acids | Carboxylic acids | Dicarboxylic acids |  |  |  |
| malic acid | Organic acids | Carboxylic acids | Dicarboxylic acids |  |  |  |
| succinic acid | Organic acids | Carboxylic acids | Dicarboxylic acids |  |  |  |
| 3-hydroxybutyric acid | Organic acids | Carboxylic acids | Hydroxycarboxylic acids | Bare | 0.616 | 0.045 |
| 4-hydroxybutyric acid | Organic acids | Carboxylic acids | Hydroxycarboxylic acids | Bare | 0.562 | 0.024 |
| aconitic acid | Organic acids | Carboxylic acids | Tricarboxylic acids |  |  |  |
| citric acid | Organic acids | Carboxylic acids | Tricarboxylic acids |  |  |  |
| 3-(3-hydroxyphenyl)propionic acid | Organic acids | Carboxylic acids |  |  |  |  |
| 4-hydroxybenzoic acid | Organic acids | Carboxylic acids |  | Bare | 0.614 | 0.005 |
| benzoic acid | Organic acids | Carboxylic acids |  |  |  |  |
| lactic acid | Organic acids | Carboxylic acids |  | Bare | 0.616 | 0.037 |
| pyrrole-2-carboxylic acid | Organic acids | Carboxylic acids |  |  |  |  |
| shikimic acid | Organic acids | Carboxylic acids |  |  |  |  |
| 2,4-diaminobutyric acid | Organic acids |  |  | Bare | 0.635 | 0.004 |
| 3-(4-hydroxyphenyl)propionic acid | Organic acids |  |  | Bare | 0.584 | 0.025 |
| phenylethylamine | Peptides | Amines | Biogenic amines |  |  |  |
| tyramine | Peptides | Amines | Biogenic amines | Bare | 0.561 | 0.008 |
| alanine | Peptides | Amino acids | Common amino acids |  |  |  |
| aspartic acid | Peptides | Amino acids | Common amino acids |  |  |  |
| glutamic acid | Peptides | Amino acids | Common amino acids |  |  |  |
| glutamine | Peptides | Amino acids | Common amino acids | Grassland | 0.718 | 0.001 |
| glycine | Peptides | Amino acids | Common amino acids |  |  |  |
| isoleucine | Peptides | Amino acids | Common amino acids | Grassland | 0.618 | 0.026 |
| leucine | Peptides | Amino acids | Common amino acids |  |  |  |
| methionine | Peptides | Amino acids | Common amino acids | Bare | 0.618 | 0.046 |
| phenylalanine | Peptides | Amino acids | Common amino acids |  |  |  |
| proline | Peptides | Amino acids | Common amino acids |  |  |  |
| serine | Peptides | Amino acids | Common amino acids |  |  |  |
| threonine | Peptides | Amino acids | Common amino acids |  |  |  |
| tyrosine | Peptides | Amino acids | Common amino acids | Grassland | 0.621 | 0.002 |
| valine | Peptides | Amino acids | Common amino acids | Grassland | 0.645 | 0.015 |
| 3-aminoisobutyric acid | Peptides | Amino acids | Other amino acids |  |  |  |
| beta-alanine | Peptides | Amino acids | Other amino acids |  |  |  |
| ornithine | Peptides | Amino acids | Other amino acids | Bare | 0.615 | 0.013 |
| oxoproline | Peptides | Amino acids | Other amino acids |  |  |  |
| glycocyamine | Peptides | Amino acids |  | Bare | 0.629 | 0.005 |
| pipecolinic acid | Phytochemical compounds | Alkaloids | Alkaloids derived from lysine |  |  |  |
| 6-hydroxynicotinic acid | Phytochemical compounds | Alkaloids | Alkaloids derived from nicotinic acid |  |  |  |
| hypoxanthine | Phytochemical compounds | Alkaloids | Others |  |  |  |
| xanthine | Phytochemical compounds | Alkaloids | Others |  |  |  |
| pinitol | Phytochemical compounds |  |  |  |  |  |
| salicylic acid | Phytochemical compounds |  |  |  |  |  |
| 4-hydroxycinnamic acid | Phytochemical compounds | Phenylpropanoids | Monolignols |  |  |  |
| ferulic acid | Phytochemical compounds | Phenylpropanoids | Monolignols |  |  |  |
| 6278 | Vitamins and Cofactors | Vitamins | Water-soluble vitamins | Grassland | 0.697 | 0.001 |
| nicotinic acid | Vitamins and Cofactors | Vitamins | Water-soluble vitamins |  |  |  |
| pantothenic acid | Vitamins and Cofactors | Vitamins | er-soluble vitamins | Bare | 0.6 | 0.01 |
| 100865 |  |  |  |  |  |  |
| 102248 |  |  |  | Bare | 0.613 | 0.014 |
| 104126 |  |  |  | Bare | 0.595 | 0.021 |
| 104312 |  |  |  |  |  |  |
| 104395 |  |  |  |  |  |  |
| 104398 |  |  |  |  |  |  |
| 104404 |  |  |  |  |  |  |
| 105209 |  |  |  | Grassland | 0.644 | 0.008 |
| 105630 |  |  |  |  |  |  |
| 106385 |  |  |  | Bare | 0.67 | 0.003 |
| 106387 |  |  |  | Grassland | 0.625 | 0.043 |
| 1064 |  |  |  | Bare | 0.68 | 0.006 |
| 106936 |  |  |  | Bare | 0.666 | 0.004 |
| 107143 |  |  |  | Bare | 0.627 | 0.01 |
| 107891 |  |  |  | Bare | 0.686 | 0.001 |
| 107941 |  |  |  |  |  |  |
| 108312 |  |  |  |  |  |  |
| 109997 |  |  |  |  |  |  |
| 110018 |  |  |  | Bare | 0.719 | 0.011 |
| 110328 |  |  |  | Grassland | 0.608 | 0.005 |
| 110343 |  |  |  |  |  |  |
| 110411 |  |  |  | Bare | 0.633 | 0.027 |
| 110573 |  |  |  |  |  |  |
| 110604 |  |  |  |  |  |  |
| 110985 |  |  |  | Bare | 0.576 | 0.027 |
| 112501 |  |  |  |  |  |  |
| 112601 |  |  |  | Grassland | 0.622 | 0.005 |
| 113510 |  |  |  |  |  |  |
| 114918 |  |  |  | Bare | 0.614 | 0.015 |
| 1173 |  |  |  | Bare | 0.664 | 0.001 |
| 118693 |  |  |  | Bare | 0.557 | 0.017 |
| 119025 |  |  |  |  |  |  |
| 119066 |  |  |  |  |  |  |
| 120526 |  |  |  | Bare | 0.648 | 0.002 |
| 120802 |  |  |  |  |  |  |
| 121473 |  |  |  | Bare | 0.602 | 0.031 |
| 124346 |  |  |  | Grassland | 0.63 | 0.002 |
| 124454 |  |  |  | Bare | 0.892 | 0.013 |
| 124484 |  |  |  |  |  |  |
| 124844 |  |  |  | Grassland | 0.625 | 0.001 |
| 124996 |  |  |  |  |  |  |
| 125784 |  |  |  | Grassland | 0.626 | 0.021 |
| 125897 |  |  |  |  |  |  |
| 126343 |  |  |  | Bare | 0.644 | 0.005 |
| 126350 |  |  |  | Grassland | 0.628 | 0.03 |
| 127343 |  |  |  |  |  |  |
| 127451 |  |  |  |  |  |  |
| 127640 |  |  |  |  |  |  |
| 127661 |  |  |  |  |  |  |
| 127676 |  |  |  |  |  |  |
| 127696 |  |  |  |  |  |  |
| 127704 |  |  |  |  |  |  |
| 129225 |  |  |  | Bare | 0.643 | 0.002 |
| 130465 |  |  |  | Bare | 0.653 | 0.019 |
| 131101 |  |  |  |  |  |  |
| 13139 |  |  |  |  |  |  |
| 132248 |  |  |  | Bare | 0.754 | 0.003 |
| 132267 |  |  |  |  |  |  |
| 133244 |  |  |  | Bare | 0.609 | 0.005 |
| 133590 |  |  |  |  |  |  |
| 133778 |  |  |  |  |  |  |
| 134 |  |  |  |  |  |  |
| 134642 |  |  |  | Bare | 0.648 | 0.001 |
| 135763 |  |  |  | Bare | 0.652 | 0.005 |
| 135777 |  |  |  |  |  |  |
| 136146 |  |  |  |  |  |  |
| 146068 |  |  |  |  |  |  |
| 146242 |  |  |  |  |  |  |
| 146259 |  |  |  |  |  |  |
| 146430 |  |  |  |  |  |  |
| 14682 |  |  |  |  |  |  |
| 14689 |  |  |  |  |  |  |
| 14724 |  |  |  | Bare | 0.623 | 0.014 |
| 160 |  |  |  |  |  |  |
| 160903 |  |  |  |  |  |  |
| 16567 |  |  |  |  |  |  |
| 16594 |  |  |  | Bare | 0.596 | 0.047 |
| 16747 |  |  |  |  |  |  |
| 16777 |  |  |  | Bare | 0.628 | 0.015 |
| 168 |  |  |  |  |  |  |
| 16833 |  |  |  | Bare | 0.622 | 0.004 |
| 1684 |  |  |  | Bare | 0.599 | 0.004 |
| 16850 |  |  |  | Bare | 0.579 | 0.009 |
| 16857 |  |  |  | Bare | 0.659 | 0.002 |
| 168799 |  |  |  | Bare | 0.617 | 0.022 |
| 168800 |  |  |  |  |  |  |
| 1704 |  |  |  |  |  |  |
| 17044 |  |  |  | Grassland | 0.571 | 0.022 |
| 17140 |  |  |  |  |  |  |
| 1725 |  |  |  |  |  |  |
| 17288 |  |  |  |  |  |  |
| 17589 |  |  |  |  |  |  |
| 17830 |  |  |  | Grassland | 0.656 | 0.004 |
| 17833 |  |  |  |  |  |  |
| 1790 |  |  |  | Bare | 0.712 | 0.006 |
| 17913 |  |  |  |  |  |  |
| 18022 |  |  |  | Bare | 0.642 | 0.014 |
| 1815 |  |  |  | Bare | 0.677 | 0.002 |
| 18305 |  |  |  | Bare | 0.6 | 0.031 |
| 183508 |  |  |  |  |  |  |
| 18386 |  |  |  | Bare | 0.653 | 0.011 |
| 18488 |  |  |  | Bare | 0.604 | 0.013 |
| 189794 |  |  |  |  |  |  |
| 1912 |  |  |  | Bare | 0.612 | 0.007 |
| 191799 |  |  |  | Grassland | 0.6 | 0.013 |
| 191801 |  |  |  |  |  |  |
| 1996 |  |  |  | Bare | 0.59 | 0.021 |
| 2-deoxytetronic acid |  |  |  | Bare | 0.573 | 0.014 |
| 2-hydroxyhexanoic acid |  |  |  |  |  |  |
| 2-hydroxyvaleric acid |  |  |  |  |  |  |
| 2-monoolein |  |  |  |  |  |  |
| 203820 |  |  |  |  |  |  |
| 2039 |  |  |  |  |  |  |
| 204582 |  |  |  |  |  |  |
| 204862 |  |  |  |  |  |  |
| 2065 |  |  |  | Bare | 0.618 | 0.008 |
| 207444 |  |  |  |  |  |  |
| 209167 |  |  |  |  |  |  |
| 209671 |  |  |  |  |  |  |
| 209675 |  |  |  |  |  |  |
| 209677 |  |  |  |  |  |  |
| 210231 |  |  |  | Bare | 0.619 | 0.003 |
| 210313 |  |  |  |  |  |  |
| 210327 |  |  |  |  |  |  |
| 210342 |  |  |  | Bare | 0.733 | 0.002 |
| 210373 |  |  |  | Bare | 0.625 | 0.009 |
| 210697 |  |  |  | Bare | 0.597 | 0.018 |
| 213019 |  |  |  |  |  |  |
| 215667 |  |  |  |  |  |  |
| 21623 |  |  |  | Bare | 0.613 | 0.005 |
| 216584 |  |  |  |  |  |  |
| 21664 |  |  |  | Bare | 0.591 | 0.005 |
| 21704 |  |  |  | Grassland | 0.571 | 0.023 |
| 21763 |  |  |  |  |  |  |
| 217691 |  |  |  |  |  |  |
| 22045 |  |  |  | Bare | 0.607 | 0.005 |
| 22064 |  |  |  | Grassland | 0.668 | 0.001 |
| 22227 |  |  |  |  |  |  |
| 2233 |  |  |  | Bare | 0.641 | 0.001 |
| 22334 |  |  |  |  |  |  |
| 22363 |  |  |  | Bare | 0.599 | 0.006 |
| 228 |  |  |  | Bare | 0.603 | 0.041 |
| 22885 |  |  |  |  |  |  |
| 22902 |  |  |  |  |  |  |
| 229977 |  |  |  |  |  |  |
| 23635 |  |  |  | Bare | 0.61 | 0.002 |
| 240031 |  |  |  |  |  |  |
| 2403 |  |  |  | Bare | 0.616 | 0.001 |
| 250544 |  |  |  |  |  |  |
| 250732 |  |  |  |  |  |  |
| 257 |  |  |  | Bare | 0.623 | 0.004 |
| 2575 |  |  |  | Bare | 0.621 | 0.015 |
| 26717 |  |  |  | Bare | 0.701 | 0.003 |
| 2684 |  |  |  | Bare | 0.713 | 0.001 |
| 2691 |  |  |  | Bare | 0.617 | 0.001 |
| 2706 |  |  |  |  |  |  |
| 2821 |  |  |  |  |  |  |
| 3-hydroxybenzoic acid |  |  |  |  |  |  |
| 3122 |  |  |  |  |  |  |
| 3206 |  |  |  |  |  |  |
| 3208 |  |  |  |  |  |  |
| 3247 |  |  |  |  |  |  |
| 324863 |  |  |  |  |  |  |
| 326249 |  |  |  | Bare | 0.71 | 0.006 |
| 326263 |  |  |  |  |  |  |
| 330511 |  |  |  |  |  |  |
| 33282 |  |  |  |  |  |  |
| 33386 |  |  |  |  |  |  |
| 33387 |  |  |  |  |  |  |
| 33395 |  |  |  |  |  |  |
| 34085 |  |  |  |  |  |  |
| 34135 |  |  |  |  |  |  |
| 341990 |  |  |  | Bare | 0.593 | 0.028 |
| 341992 |  |  |  |  |  |  |
| 342183 |  |  |  |  |  |  |
| 342561 |  |  |  | Bare | 0.598 | 0.031 |
| 342919 |  |  |  |  |  |  |
| 345265 |  |  |  | Bare | 0.617 | 0.005 |
| 346225 |  |  |  |  |  |  |
| 346241 |  |  |  | Bare | 0.663 | 0.005 |
| 346242 |  |  |  |  |  |  |
| 346248 |  |  |  |  |  |  |
| 3465 |  |  |  |  |  |  |
| 347514 |  |  |  |  |  |  |
| 347755 |  |  |  | Bare | 0.613 | 0.008 |
| 348828 |  |  |  |  |  |  |
| 349293 |  |  |  |  |  |  |
| 357024 |  |  |  |  |  |  |
| 359832 |  |  |  |  |  |  |
| 360226 |  |  |  |  |  |  |
| 360824 |  |  |  |  |  |  |
| 360825 |  |  |  | Bare | 0.649 | 0.036 |
| 360827 |  |  |  |  |  |  |
| 360842 |  |  |  |  |  |  |
| 360846 |  |  |  | Bare | 0.6 | 0.01 |
| 360852 |  |  |  |  |  |  |
| 365122 |  |  |  |  |  |  |
| 365741 |  |  |  |  |  |  |
| 365772 |  |  |  |  |  |  |
| 366259 |  |  |  | Bare | 0.631 | 0.031 |
| 367449 |  |  |  |  |  |  |
| 370223 |  |  |  | Grassland | 0.682 | 0.009 |
| 371262 |  |  |  |  |  |  |
| 371566 |  |  |  |  |  |  |
| 371571 |  |  |  | Bare | 0.615 | 0.002 |
| 376594 |  |  |  | Bare | 0.625 | 0.019 |
| 376621 |  |  |  |  |  |  |
| 376622 |  |  |  |  |  |  |
| 376631 |  |  |  |  |  |  |
| 376647 |  |  |  | Bare | 0.613 | 0.018 |
| 376854 |  |  |  |  |  |  |
| 376958 |  |  |  |  |  |  |
| 377041 |  |  |  | Bare | 0.627 | 0.019 |
| 377232 |  |  |  |  |  |  |
| 377271 |  |  |  |  |  |  |
| 377308 |  |  |  |  |  |  |
| 377341 |  |  |  |  |  |  |
| 377405 |  |  |  |  |  |  |
| 377766 |  |  |  |  |  |  |
| 377770 |  |  |  | Grassland | 0.816 | 0.009 |
| 377949 |  |  |  | Bare | 0.574 | 0.028 |
| 378059 |  |  |  | Bare | 0.64 | 0.009 |
| 3781 |  |  |  | Grassland | 0.569 | 0.011 |
| 378176 |  |  |  | Bare | 0.624 | 0.003 |
| 378198 |  |  |  | Grassland | 0.644 | 0.012 |
| 378294 |  |  |  | Bare | 0.593 | 0.018 |
| 378468 |  |  |  |  |  |  |
| 378531 |  |  |  |  |  |  |
| 378606 |  |  |  | Bare | 0.576 | 0.028 |
| 378763 |  |  |  | Bare | 0.709 | 0.001 |
| 379060 |  |  |  |  |  |  |
| 379063 |  |  |  |  |  |  |
| 379095 |  |  |  | Bare | 0.591 | 0.022 |
| 379168 |  |  |  |  |  |  |
| 379197 |  |  |  | Bare | 0.594 | 0.003 |
| 379411 |  |  |  |  |  |  |
| 379527 |  |  |  |  |  |  |
| 379636 |  |  |  | Bare | 0.605 | 0.048 |
| 379787 |  |  |  |  |  |  |
| 380100 |  |  |  | Bare | 0.706 | 0.006 |
| 380254 |  |  |  | Bare | 0.597 | 0.024 |
| 380511 |  |  |  |  |  |  |
| 381429 |  |  |  |  |  |  |
| 381497 |  |  |  | Bare | 0.764 | 0.022 |
| 39801 |  |  |  |  |  |  |
| 4',5-dihydroxy-7-glucosyloxyflavanone |  |  |  | Grassland | 0.635 | 0.009 |
| 41811 |  |  |  |  |  |  |
| 41821 |  |  |  |  |  |  |
| 41836 |  |  |  | Bare | 0.591 | 0.028 |
| 41882 |  |  |  |  |  |  |
| 41924 |  |  |  |  |  |  |
| 42161 |  |  |  | Bare | 0.627 | 0.036 |
| 43734 |  |  |  |  |  |  |
| 4526 |  |  |  |  |  |  |
| 4550 |  |  |  |  |  |  |
| 46131 |  |  |  |  |  |  |
| 46281 |  |  |  | Bare | 0.62 | 0.008 |
| 46346 |  |  |  |  |  |  |
| 4709 |  |  |  |  |  |  |
| 473 |  |  |  |  |  |  |
| 47358 |  |  |  | Bare | 0.644 | 0.014 |
| 4766 |  |  |  |  |  |  |
| 479 |  |  |  |  |  |  |
| 48427 |  |  |  |  |  |  |
| 49426 |  |  |  | Bare | 0.627 | 0.01 |
| 51865 |  |  |  |  |  |  |
| 5244 |  |  |  |  |  |  |
| 53737 |  |  |  | Bare | 0.574 | 0.022 |
| 54 |  |  |  | Grassland | 0.669 | 0.002 |
| 6104 |  |  |  | Bare | 0.664 | 0.001 |
| 62250 |  |  |  | Bare | 0.61 | 0.045 |
| 66311 |  |  |  |  |  |  |
| 72488 |  |  |  |  |  |  |
| 7408 |  |  |  |  |  |  |
| 84116 |  |  |  | Bare | 0.686 | 0.005 |
| 84181 |  |  |  |  |  |  |
| 84193 |  |  |  |  |  |  |
| 85168 |  |  |  | Bare | 0.604 | 0.023 |
| 8598 |  |  |  | Grassland | 0.719 | 0.002 |
| 87834 |  |  |  |  |  |  |
| 87951 |  |  |  | Bare | 0.622 | 0.003 |
| 88501 |  |  |  | Bare | 0.598 | 0.012 |
| 88847 |  |  |  |  |  |  |
| 89145 |  |  |  |  |  |  |
| 89252 |  |  |  |  |  |  |
| 91421 |  |  |  |  |  |  |
| 93947 |  |  |  |  |  |  |
| 97743 |  |  |  | Bare | 0.614 | 0.01 |
| 98101 |  |  |  |  |  |  |
| 99 |  |  |  |  |  |  |
| acetophenone |  |  |  |  |  |  |
| allantoic acid |  |  |  |  |  |  |
| azelaic acid |  |  |  |  |  |  |
| beta sitosterol |  |  |  | Grassland | 0.669 | 0.012 |
| beta-mannosylglycerate |  |  |  | Bare | 0.572 | 0.039 |
| cerotinic acid |  |  |  | Bare | 0.616 | 0.01 |
| citraconic acid |  |  |  |  |  |  |
| conduritol-beta-epoxide |  |  |  |  |  |  |
| ethanolamine |  |  |  |  |  |  |
| heptadecanoic acid |  |  |  |  |  |  |
| hexuronic acid |  |  |  |  |  |  |
| hydrocinnamic acid |  |  |  | Bare | 0.591 | 0.05 |
| levoglucosan |  |  |  | Bare | 0.675 | 0.003 |
| methylmalonic acid |  |  |  |  |  |  |
| octadecanol |  |  |  | Bare | 0.596 | 0.022 |
| oleamide |  |  |  |  |  |  |
| palatinitol |  |  |  | Grassland | 0.598 | 0.005 |
| parabanic acid |  |  |  |  |  |  |
| pentose |  |  |  | Bare | 0.684 | 0.001 |
| phosphoethanolamine |  |  |  | Grassland | 0.73 | 0.004 |
| phytanic acid |  |  |  | Bare | 0.613 | 0.016 |
